# An oxidorhenium(v) complex with an electron-withdrawing ligand: benefits and drawbacks for a dual role catalyst†

**DOI:** 10.1039/d4ra07391f

**Published:** 2024-12-23

**Authors:** A. Gradenegger, J. A. Schachner, F. Belaj, N. C. Mösch-Zanetti

**Affiliations:** a Institute of Chemistry, University of Graz Schubertstraße 1 8010 Graz Austria joerg.schachner@uni-graz.at

## Abstract

One very unique feature of oxidorhenium(v) complexes is their dual catalytic activity in both reduction of stable oxyanions like perchlorate ClO_4_^−^ and nitrate NO_3_^−^ as well as epoxidation of olefins. In our ongoing research efforts, we were interested to study how an electron-withdrawing ligand would affect both these catalytic reactions. Hence, we synthesized the novel bidentate dimethyloxazoline-dichlorophenol ligand HL1 and synthesized oxidorhenium(v) complex [ReOCl(L1)_2_] (1). Then, catalytic experiments were conducted showing that non-redox epoxidation activity is indeed enhanced, but redox catalysis *via* oxygen atom transfer (OAT) activity was reduced for ClO_4_^−^ and NO_3_^−^ reductions. From one nitrate reduction experiment, a small amount of the singly-oxidized dioxidorhenium(vi) complex [ReO_2_(L1)] (2) could be isolated, confirming the successful reduction sequence of nitrate to nitrite NO_2_^−^ (2e^−^ reduction) to NO (1e^−^ reduction). Furthermore, ligand L1 displayed a richer than usually observed coordination chemistry, allowing for the isolation of complexes [ReOCl_2_(SMe_2_)(L1)] (*trans*-3a), [ReOCl_2_(OPPh_3_)(L1)] (3b) and [ReCl_3_(OPPh_3_)(L1)] (3c). Complexes 1 and 3a-b were tested in cyclooctene epoxidation, 1 was additionally investigated as an oxyanion reduction catalyst of perchlorate and nitrate. All compounds HL1, 1, 2 and 3a–c could be characterized by single-crystal X-ray diffraction, besides other routine analyses.

## Abstract 2

Electron-withdrawing ligands increase epoxidation activity, but decrease oxyanion reduction catalysis.

## Graphical abstract

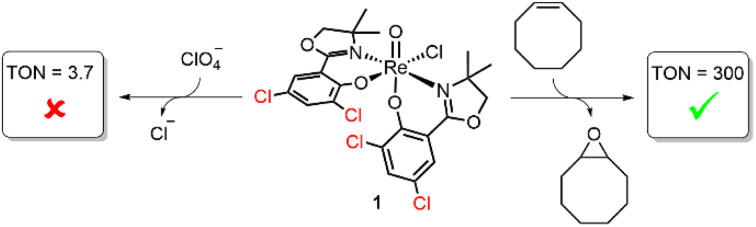

## Introduction

High oxidation state rhenium chemistry stepped into the spotlight with the breakthrough discovery by Herrmann and co-workers of methyltrioxorhenium (MTO) and its high catalytic activity in olefin epoxidation chemistry in 1991.^1^ Soon thereafter, oxidorhenium(v) complexes were investigated as potentially less sensitive, more group tolerant alternatives to MTO.^2–4^ Other catalytic applications were found in (enantio)selective reduction of ketones and imines,^5^ or the activation of small molecules like O_2_, CO (ref. 6) or CH_4_.^7,8^ Recently, various pincer complexes of Re showed activity in the splitting of dinitrogen N_2_.^9^ Moreover it was shown that the Lewis-basic oxido ligand in oxidorhenium(v) complexes can be triggered by the frustrated Lewis-pair concept to take part in catalytic reactions, that are otherwise not occurring. Hence, an NNN-pincer complex of oxidorhenium(v) showed hydrogenation activity of olefins only when activated by B(C_6_F_5_)_3_.^10^ Beside these catalytic applications, oxidorhenium(v) complexes are more and more investigated as potential metallodrugs for chemotherapeutical applications.^11^ Due to the robustness of the high oxidation states of rhenium complexes, such complexes were also investigated in oxygen atom transfer (OAT) catalysis, where several parallels to Mo-containing enzymes were identified.^8,12,13^

**Fig. 1:**
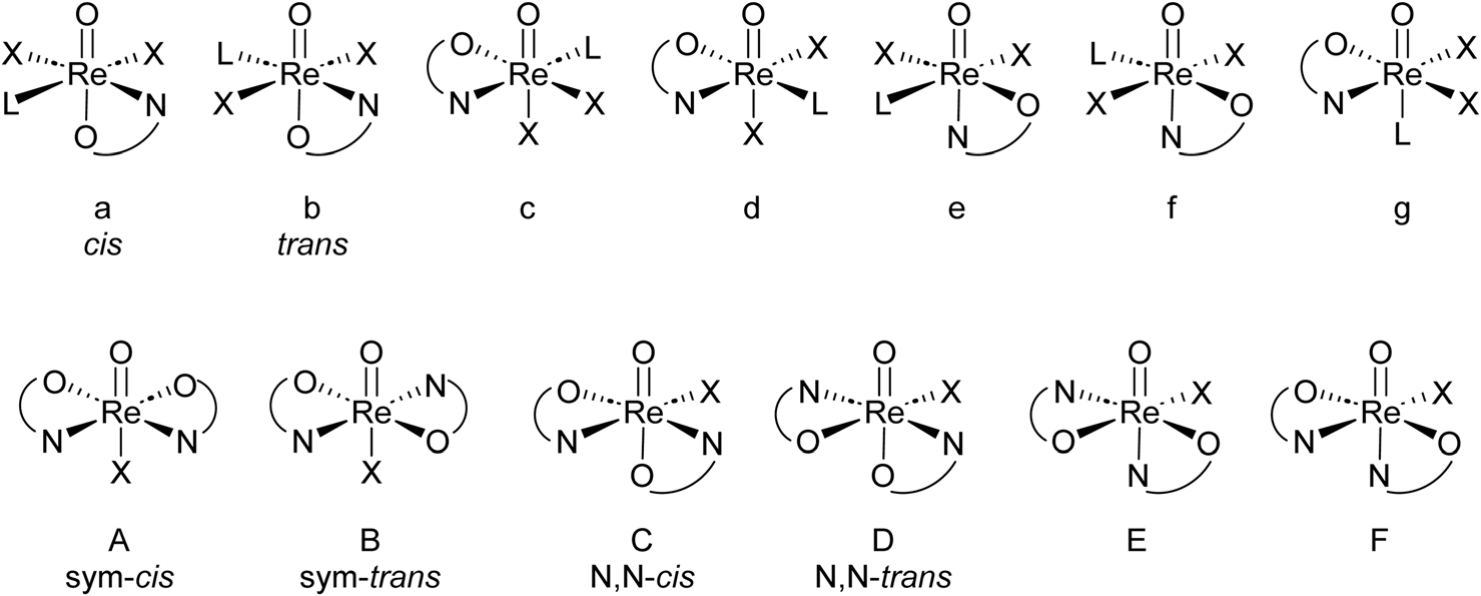
Top: possible stereoisomers a–g for mono-ligated complexes; bottom: possible stereoisomers A–F for bis-ligated complexes.

Oxidorhenium(v) complexes show a rich coordination chemistry towards a variety of bidentate ligands. Several reviews covering the extensive structural chemistry of these complexes have been published by Sergienko and Machura.^4,14–16^ There are, in principle, seven possible stereoisomers for mono-ligated complexes of type [ReOX_2_L(L_ON_)] and six for bis-ligated complexes of type [ReOX(L_ON_)_2_] (L = neutral, mono-dentate ligand; X = mono-anionic, mono-dentate ligand; L_ON_ = mono-anionic, bidentate ON-ligand).^17^ An overview of both possible sets of stereoisomers is given in Fig. 1.

For mono-ligated isomers a and b, the literature established *cis*/*trans* labels refer to the orientation of the two X ligands. Of the six bis-ligated isomers A–F, two show a higher symmetry (*C*_*s*_ for A; *C*_2_ for B) than the four *C*_1_-symmetric isomers C–F. Therefore, isomers A and B are referred to as *sym*–*cis* and *sym*–*trans* and isomers C and D as *N*,*N-cis*/*trans*, with the *cis*/*trans* labels referring to the position of the nitrogen atoms of the L_ON_-ligands. Isomers E and F have not been isolated yet in oxidorhenium(v) chemistry, which is due the strong *trans*-influence of the oxido ligand.^4^ Hence, examples of bis-ligated stereoisomers E and F, where the neutral nitrogen donor would be *trans* to the oxido ligand, have not been isolated yet. An interconversion of one isomer into the other has not been reported yet. Hence, they are considered to be static. Isomers B–F are in addition enantiomeric.

**Fig. 2:**
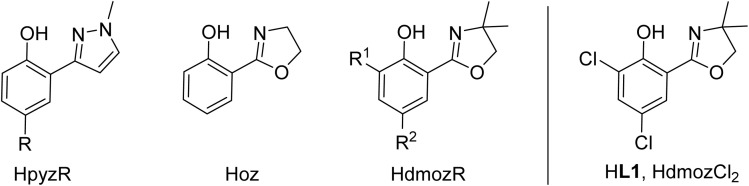
Previously established bidentate HL_ON_ ligands in oxidorhenium(v) chemistry with commonly used abbreviations (HpyzR; Hoz; HdmozR); new ligand HdmozCl_2_ (HL1) investigated herein.

Oxidorhenium(v) complexes also belong to a rare class of catalysts capable of reducing oxyanions like perchlorate ClO_4_^−^ and nitrate NO_3_^−^. Only few other metal complexes are able to catalytically reduce perchlorate.^18,19^ The initial discovery of rhenium-catalyzed perchlorate reduction was disclosed by Abu-Omar and co-workers in 2000 and subsequently studied in detail.^20–22^ By using the dihydro-oxazoline-phenol ligand Hoz (Fig. 2) in complex [ReOCl(oz)_2_] and 4 equiv. of SMe_2_ as sacrificial reductant, perchlorate ClO_4_^−^ could be fully reduced to chloride Cl^−^ in four sequential OAT steps with a catalyst loading of 3.2 mol% under ambient conditions (25 °C, CH_3_CN/H_2_O = 95/5 vol%). The rate determining step was identified as the first OAT step yielding chlorate ClO_3_^−^.^21,22^ This intriguing reaction also sparked our interest, and we could show that only bis-ligated complexes show activity in perchlorate reduction.^23,24^ Stereoisomerism plays an important role, as the *N*,*N-trans* isomer D shows superior activity over the *N*,*N-cis* isomer C.^24^ Further advances for a possible application in waste water treatment of perchlorate were made by Strathmann and Liu.^25^ Catalytic nitrate reduction, which would be highly desirable in the context of removal of water contaminants, is the newest research field for oxidorhenium(v) complexes.^26^ Literature precedence for complexes that do show nitrate reduction activity is scarce, and often these systems are not catalytic, or only function under strict exclusion of air and moisture.^19,27^ Here, one challenge is to selectively reduce NO_3_^−^ to N_2_, not NH_3_, which requires five electrons, and therefore a catalyst that must be capable of performing both single- and two-electron reduction steps.^28^

**Scheme 1:**
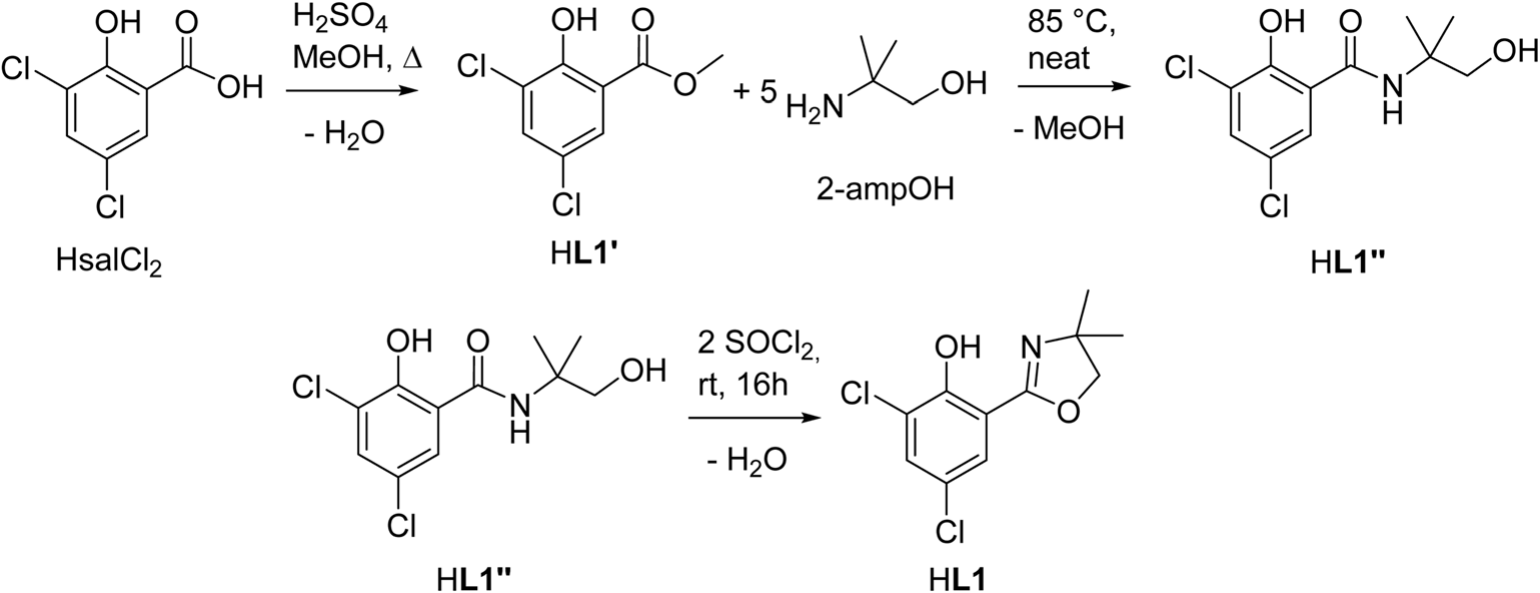
Three-step synthesis of ligand HL1.

**Scheme 2:**
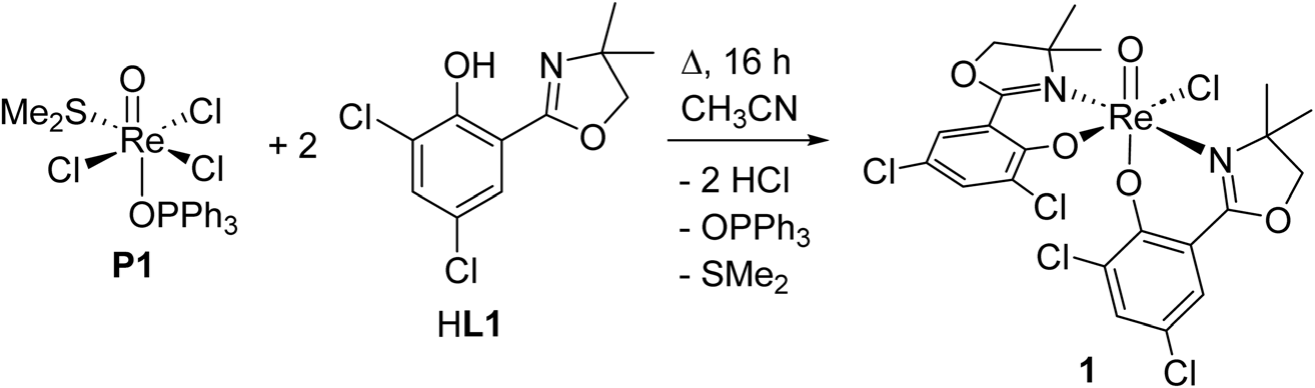
Successful reaction conditions (16 h) for synthesis of oxidorhenium(v) complex [ReOCl(L1)_2_] (1).

**Scheme 3:**
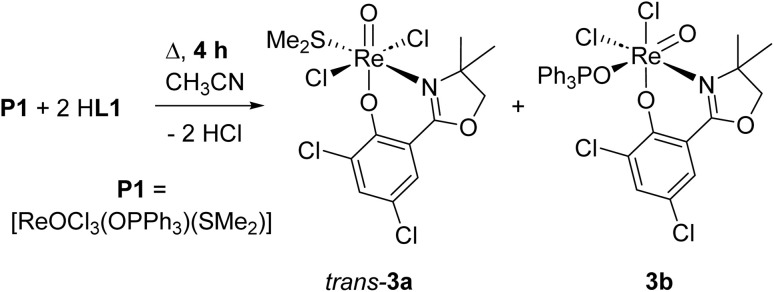
Formation of oxidorhenium(v) complexes *trans*-3a and 3b under too short reaction time.

Within this manuscript, the synthesis of electron-withdrawing ligand HL1 (Scheme 1, synthesis details and solid-state structure in ESI†), and its coordination chemistry are presented. The successful synthesis of targeted complex [ReOCl(L1)_2_] (1) (Scheme 2), the formation of dioxidorhenium(vi) complex [ReO_2_(L1)_2_] (2, Scheme 6) from a catalytic nitrate reduction experiment and the isolation of three side-products, those are mono-ligated complexes [ReOCl_2_(SMe_2_)(L1)] (*trans*-3a), [ReOCl_2_(OPPh_3_)(L1)] (3b) (Scheme 3) and the reduced rhenium(IV) complex [ReCl_3_(OPPh_3_)(L1)] (3c) (see ESI†), are described. The influence of the electron-withdrawing chlorido substituents was evaluated in catalytic epoxidation of cyclooctene and oxyanion reduction reactions.

## Results and discussion

### Synthesis of ligand HL1

The optimized three-step synthesis of ligand HdmozCl_2_ (HL1, Scheme 1, for details see ESI†) does not require column chromatography. Starting from the respective salicylic acid, first methyl ester HL1′ is quantitatively synthesized by boiling HsalCl_2_ in MeOH in presence of sulfuric acid. The so obtained methyl ester can be cleanly turned with high yields (>95%) into the benzamide HL1′′ with 2-methyl-2-amino-propanol (2-ampOH) under neat conditions. The final ring closure to HL1 is achieved with SOCl_2_, also giving high yields (>95%). The synthesis of HL1 was also attempted under microwave heating. However, the obtained yields and purities by conventional heating were always higher compared to microwave heating (Tables S1 and S2†). As all three steps are condensation reactions with H_2_O or MeOH being eliminated (Scheme 1), the observed higher yields might simply be explained by use of an open-vessel reflux condenser, compared to the closed-system, pressurized microwave heating (for details see ESI†).

### Synthesis and isolation of rhenium complexes

**Fig. 3:**
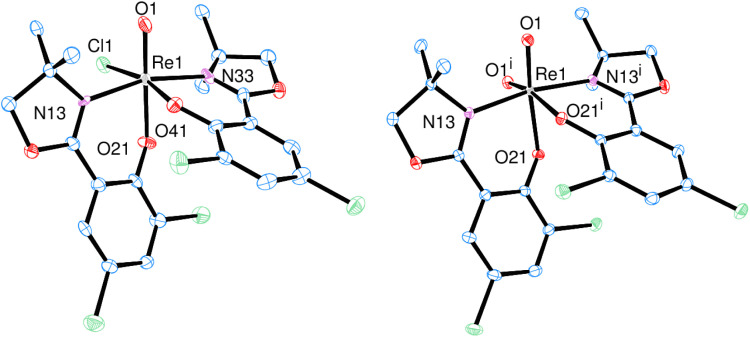
Molecular views (50% level) of oxidorhenium(v) complex 1 (left) and dioxidorhenium(vi) complex 2 (right) (H atoms and solvent molecules omitted for clarity).

Bis-ligated complex [ReOCl(L1)_2_] (1) was obtained by reacting two equivalents of HL1 with precursor [ReOCl_3_(OPPh_3_)(SMe_2_)] (P1) under refluxing conditions of a minimum of 16 h in CH_3_CN (Scheme 2). Crude, green material of 1 was purified by several re-crystallizations from CH_2_Cl_2_/heptane to give a final yield of 45%. The so obtained green crystalline material shows the expected *C*_1_-symmetric ^1^H NMR spectrum for the two ligand moieties (Fig. S6†). Complex 1 is rather insoluble in apolar solvents and moderately soluble in polar solvents like CH_2_Cl_2_, CHCl_3_ and CH_3_CN. The Re=O bond is located at 975 cm^−1^ in the IR spectrum and the mass spectrum shows the M^+^ peak at 756.4 with the correct isotope pattern. In the solid state, 1 adopts an *N*,*N-trans* isomeric form (left, Fig. 3), consistent with other published rhenium complexes employing an HdmozR ligand.^23,24^

From one nitrate reduction experiment with pre-catalyst 1 (*vide infra*, Scheme 6), after the 24 h reaction time, a small amount (app. 1.5 mg) of orange crystals had formed in the reaction mixture, in contrast to the green color of complex 1. The crystallinity was high enough to allow for analysis by single-crystal X-ray diffraction. These orange crystals revealed to be the dioxidorhenium(vi) complex [ReO_2_(L1)_2_] (2) (right, Fig. 3). Because of the small amount of isolated material, no further characterization was possible. Complex 2 is the product of a single electron reduction of nitrite NO_2_^−^ to NO under concomitant oxidation of oxidorhenium(v) complex 1 to give paramagnetic dioxidorhenium(vi) complex 2 (Scheme 6). If a mixture of P1 and two equivalents of HL1 is reacted for only four hours under boiling conditions, mixtures of complexes *trans*-3a and 3b are obtained (Scheme 3). They could be isolated based on their different solubilities. Details on formation and separation of complexes 3a and 3b as well as on the solid state structure of decomposition product [ReCl_3_(OPPh_3_)(L1)] (3c) can be found in the ESI.†

### Molecular structures

Single crystals of [ReOCl(L1)_2_] (1) were grown from a saturated CH_2_Cl_2_ solution layered with heptane, complex [ReO_2_(L1)_2_] (2) crystallized upon cooling a reaction mixture (CH_3_CN/H_2_O = 50/50 vol%) from 50 °C to room temperature (Fig. 3).

Both complexes adopt the *N*,*N-trans* isomeric form in the solid state. The rhenium centers are coordinated in a distorted octahedral fashion. The presence of a second oxido ligand in dioxidorhenium(vi) complex 2 however causes significant structural deviations from an octahedron. The distances of the oxido bonds are elongated in 2 compared to 1 (Table 1). The angle O1-Re-O1^i^ between the two oxido ligands is 108.65(8)°, which strongly deviates from ideal 90°. This widening of the dioxide angle results in a closer proximity of the two L1 ligand moieties. The angle of the least square planes between the two phenyl rings is only 36.09(8)°. A comparison with previously published oxidorhenium(v) and dioxidorhenium(vi) complexes shows that almost all bond lengths are the same within experimental error (see Tables S3 and S4†). A notable exception is complex [ReOCl(dmoz)_2_], which still shows the longest Re-Cl1 bond length of 2.440(2) Å (ref. 23) of all published [ReOCl(dmozR)_2_] complexes so far. As perchlorate and nitrate reduction catalysis operates under a dissociative mechanism (Scheme 6), the Re-Cl1 bond length is probably related to catalytic activity.

**Table 1:** Selected bond lengths [Å] of oxidorhenium(v) complex 1 and dioxidorhenium(vi) complex 2

	Re1=O1	Re–Cl1/O1^a^	Re–O21	Re1–O41	Re–N13	Re–N33
1	1.700(5)	2.3960(17)	2.016(4)	2.006(4)	2.106(5)	2.096(5)
2	1.7397(13)	1.7397(13)^a^	2.0680(12)	2.0680(12)^a^	2.0963(14)	2.0963(14)^a^

a Equivalent atoms are generated by symmetry (two-fold rotation axis).

**Fig. 4:**
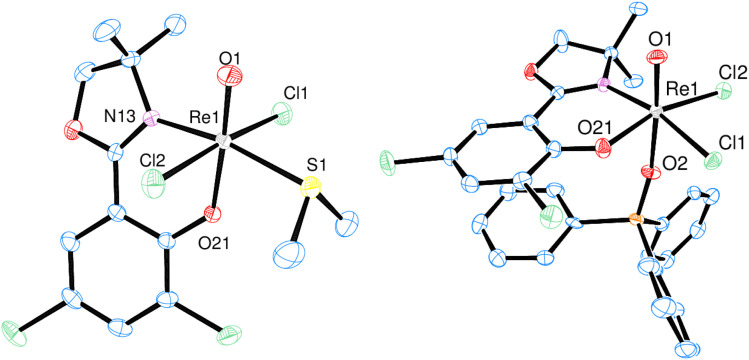
Molecular views (50% level) of complexes *trans*-3a (left) and 3b (right) (H atoms and solvent molecules omitted for clarity).

Single-crystals of *trans*-3a and 3b were obtained by re-crystallization from saturated acetonitrile solutions. The molecular structures are displayed in Fig. 4. In both cases, the rhenium center is coordinated in a distorted octahedron. In the solid state, *trans*-3a adopts a *trans*-dichlorido isomer, with the phenolate oxygen atom O21 *trans* to the oxido ligand and the two chlorido ligands *trans* to each other. In contrast, 3b adopts an isomeric form where the OPPh_3_ ligand is *trans* to the oxido ligand, and L1 is coordinated in the equatorial plane. The two chlorido ligands are in a *cis* position to each other, completing the octahedral coordination of 3b. Such an isomer, with an OPPh_3_ ligand *trans* to the oxido ligand, had been isolated once before.^29^ The bond angles for 3a and 3b are all within the expected range and show small deviations from a perfect octahedron.^15^ In *trans*-3a, the Cl1–Re1–Cl2 angle is 171.43(5)°, in 3b the O1–Re1–O2 angle is 173.90(12)°. Selected bond lengths for *trans*-3a and 3b can be found in Table 2, the full list for 3a in the ESI.† Only six other structurally characterized rhenium complexes containing the SMe_2_ ligand have been reported in literature, one of them being the used precursor [ReOCl_3_(OPPh_3_)(SMe_2_)] (P1) itself.^13,30^ The respective bond distances between P1 and *trans*-3a are the same within experimental error. For example the Re–S bond in P1 is 2.425(2) Å, in *trans*-3a Re1–S1 is 2.4238(15) Å. In 3b, the Re1=O1 bond distance of 1.692(3) Å is significantly elongated compared to P1 at 1.651(4) Å.

**Table 2:** Selected bond lengths [Å] of *trans*-3a and 3b

	Re1=O1	Re1–O21	Re1–Cl1	Re–Cl2	Re–S1/O2	Re–N13
*trans*-3a	1.677(4)	1.989(3)	2.3846(15)	2.4057(14)	2.4238(15)	2.113(5)
3b	1.692(3)	1.998(2)	2.3670(9)	2.3858(10)	2.141(3)	2.107(3)

### Cyclic voltammetry

To study the electron-withdrawing effect of ligand moiety L1 on the rhenium(v) center, complexes [ReOCl(L1)_2_] (1), [ReOCl_2_(L1)(SMe_2_)] (3a) and [ReOCl_2_(L1)(OPPh_3_)] (3b) were investigated by cyclic voltammetry, but only 1 and 3a showed a quasi-reversible Re(v)/Re(vi) redox couple at positive potentials, which proved to be stable over several cycles and independent of scan-rate. A standard three electrode setup in CH_3_CN under inert conditions was used, with analyte solutions near 1 mM with (NBu_4_)PF_6_ as supporting electrolyte (0.1 M). The currents *I*_p_ were normalized by the actual concentrations to allow for better comparability. Half-wave potentials *E*_1/2_ (*E*_1/2_ = (*E*_p,c_ + *E*_p,a_)/2) are given in Table 3.

**Table 3:** Redox potentials *E*_1/2_ [V] of complexes 1 and 3a at 200 mV s^−1^

	1	3a
*E*_1/2_ [V]	0.85	1.13

The influence of the electron-withdrawing ligand L1 is reflected in the shift of the Re(v)/Re(vi) redox couple to higher potential. The redox couple of unsubstituted complex [ReOCl(dmoz)_2_] is at 0.64 V.^23^ This confirms a more electron-poor rhenium center when ligand L1 is coordinated. Mono-ligated complex 3a is shifted the most to higher potential, 280 mV higher than bis-ligated complex 1. The shift to 1130 mV for 3a implies a very electron-poor Re center, potentially due to the presence of two chlorido ligands and an electron-withdrawing L1 moiety. Such a quasi-reversible redox couple for a mono-ligated complex like 3a is rarely observed. For many other oxidorhenium(V) complexes of the type [ReOCl_2_(L)(L_ON_)] (L usually is PPh_3_), only irreversible faradaic processes could be recorded by cyclic voltammetry, due to decomposition under the experimental conditions.^23,31,32^

### Epoxidation catalysis

**Scheme 4:**
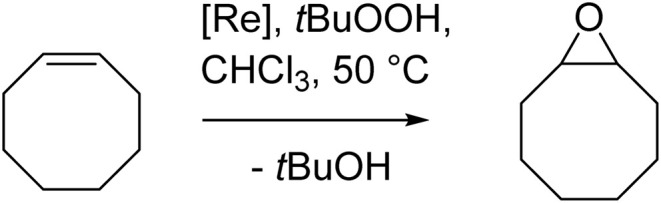
Catalytic epoxidation of cyclooctene.

Complexes 1 and 3a-b were tested in cyclooctene epoxidation using *tert*-butylhydroperoxide (TBHP, 5.5 M in decane) as the oxidant (CHCl_3_, 50 °C, 1 mol% catalyst loading, 3 equiv. TBHP). Aliquots were withdrawn at given time intervals and the conversion to epoxide analyzed by GC/MS (Scheme 4).

**Fig. 5:**
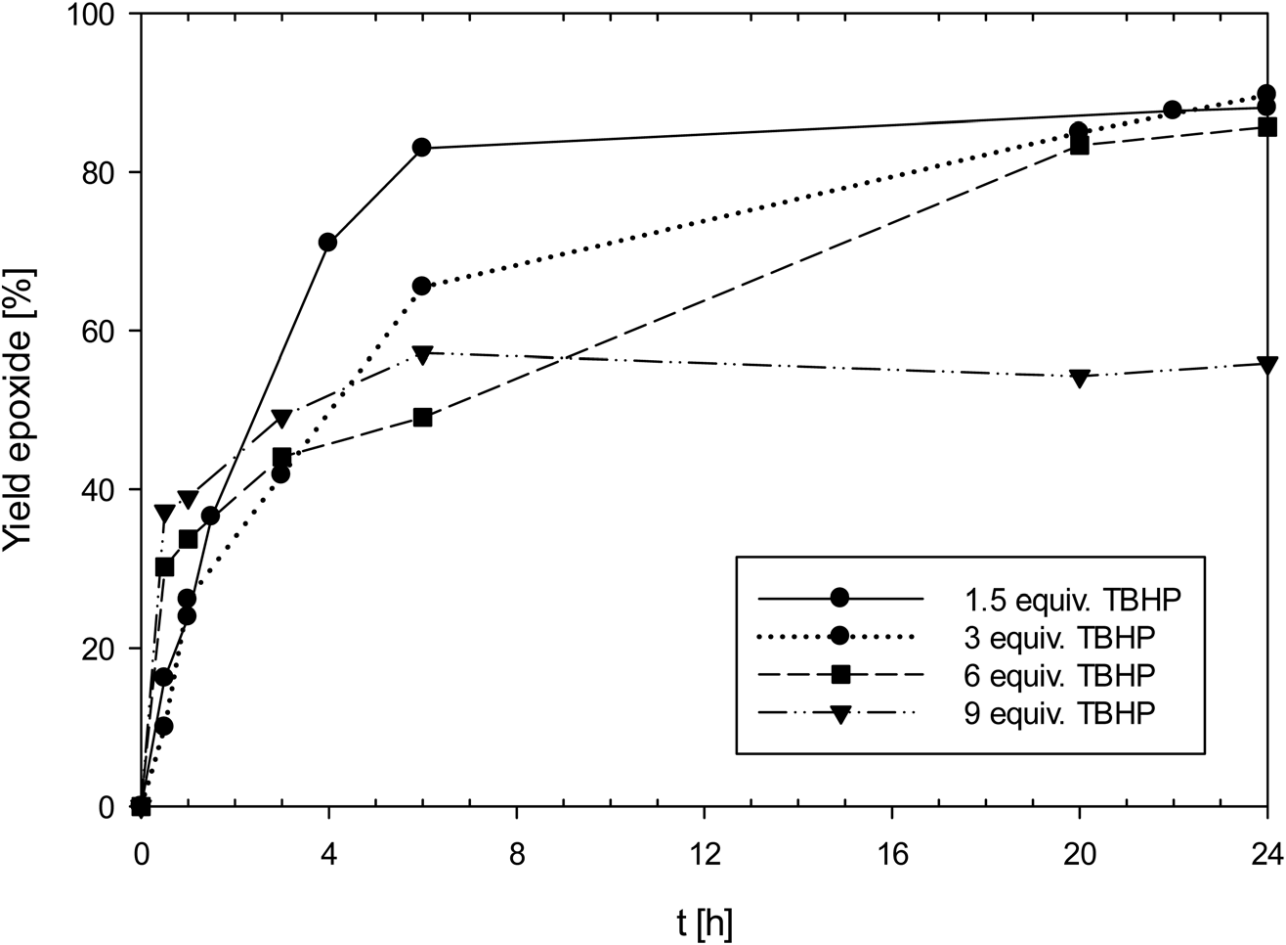
Yield of epoxide with catalyst 1 with different amounts of TBHP (50 °C, CHCl_3_, 1 mol% 1; equiv. TBHP = molar equivalents to cyclooctene; lines between measured points are just optical guidelines without physical meaning).

Especially complex 1 showed high activities for an oxidorhenium(v) complex of the type [ReOCl(L_ON_)_2_] (*vide infra*), reaching a TON of 300 at 0.1 mol% catalyst loading. A summary of results for 1 is shown in Table 4. Complex 1 was also tested for the potential phenomenon of “dilution effect”, that was recently described for Mo and W epoxidation catalysts.^33^ The dilution effect describes the phenomenon where catalyst activity is low at 1 mol% loading, but increases significantly at lower catalyst loadings, seemingly at higher dilution. The low ratio of catalyst to TBHP at 1 mol% loading (1 : 300) causes this low activity, and it was shown that by increasing the amount of TBHP, the same catalyst shows a much-improved activity at the same 1 mol% loading. In order to test this hypothesis, the effect of the catalyst to TBHP ratio at 1 mol% catalyst loading was investigated (Fig. 5). In addition to the standard conditions (3 equiv. of TBHP) experiments with 1.5, 6 and 9 equivalents of TBHP were conducted (entries 2–4, Table 4). In contrast to the above mentioned “dilution effect”, with 1, the decrease to 1.5 equivalents of TBHP seemed to even have a beneficial effect, as in this experiment, a higher initial productivity was observed. In contrast, at 9 equivalents of TBHP, the yield of epoxide only reached 56%, under otherwise the same conditions (50 °C, CHCl_3_, 1 mol% 1). It therefore seems that 1 is not suffering from the “dilution effect”, but that higher amounts of TBHP are rather detrimental to catalyst activity due to faster oxidation to unreactive perrhenate (*vide infra*).

**Table 4:** Summary of epoxidation activity for complex 1 and comparison to previously published catalysts^a^

Entry	Cat.	mol%	Equiv. TBHP	TON []	TOF [h^−1^]
1	1	1	3	90	3.75
2	1	1	1.5	89	3.71
3	1	1	6	82	3.42
4	1	1	9	56	2.3
5	1	0.5	3	180	7.5
6	1	0.1	3	300	12.5
7	ReOCl(dmozNO_2_)_2_ (ref. 23)	1	3	80	9.4
8	ReOCl(pyzBr)_2_ (ref. 31)	1	3	97	32
9	ReOCl(pyzOMe)_2_ (ref. 31)	1	3	92	31
10	ReOCl(pyzOMe)_2_ (ref. 31)	0.1	3	200	8.3

a Reaction conditions: 50 °C, CHCl_3_; TON were calculated at maximum conversion to epoxide; TOF were calculated for the time at maximum conversion to epoxide.

**Fig. 6:**
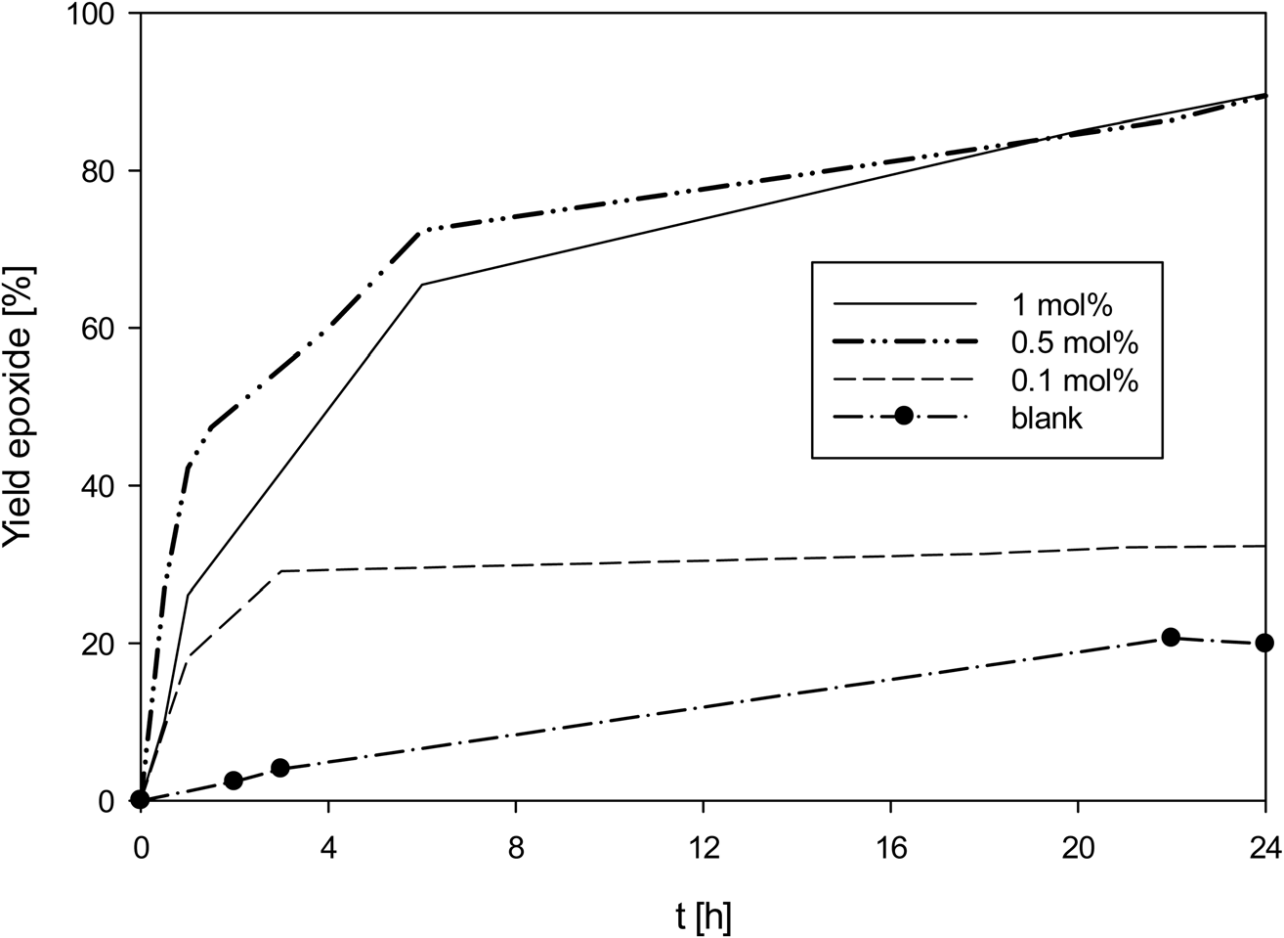
Yield of epoxide with catalyst 1 at different catalyst loadings (50 °C, CHCl_3_, 3 equiv. TBHP; lines between measured points are just optical guidelines without physical meaning).

As complex 1 performed rather well for an oxidorhenium(v) complex at 1 mol%, also lower catalyst loadings of 0.5 and 0.1 mol% were investigated (Fig. 6). At 0.5 mol% catalyst loading, again almost full conversion (90%) of cyclooctene to epoxide was reached, resulting in a TON of 180 (entry 5, Table 4). And even at 0.1 mol%, still a conversion to 30% epoxide was reached, resulting in a TON of 300 (entry 6, Table 4). To confirm the epoxidation is still catalyzed by 1, a blank experiment under the otherwise same conditions was done. As shown in Fig. 6, app. 20% of the cyclooctene were converted over 24 h, slightly below the 30% conversion with 1 at 0.1 mol%. In the uncatalyzed reaction however, a linear increase of epoxide is observed, whereas the rhenium catalyzed reactions show the typical kinetic profiles for a metal catalyzed reaction. The loss of activity of 1 after 3 h points to a decomposition of the catalyst, most likely by oxidation to inactive perrhenate. The complete discoloration of the reaction mixture supports this conclusion.^3,32^ Lowering the amount of TBHP from 3 to 1.5 molar equivalents did not improve catalyst activity at 0.1 mol% catalyst loading.

Compared to our previously tested phenol-oxazoline [ReOCl(dmozR)_2_]^23^ complexes, with a TON reaching 300, complex 1 proved to be highly active (Table 4). It thereby succeeded all of our previously investigated complexes of the HdmozR ligand set. Under the same conditions, the next best activity (TON = 80) was observed for complex [ReOCl(dmozNO_2_)_2_].^23^ Only the related phenolate–pyrazole complexes [ReOCl(pyzR)_2_] showed even higher turnover numbers of TON = 92–97.^31^ The first tested oxidorhenium(v) complexes in 1995, equipped with Schiff base ligands, showed TONs around 70 under similar conditions (1 mol% catalyst, CHCl_3_, 50 °C, 1 equiv. TBHP).^34^ A review summarizing the epoxidation activity of oxidorhenium(v) catalysts up to 2014 was published by Machura and co workers, showing activities between 0 to 75 turnovers.^4^ To the best of our knowledge, there were no other reported complexes with higher activities. Both for the [ReOCl(dmozR)_2_] (R = Cl or NO_2_) as well as the [ReOCl(pyzR)_2_] (R = Br or NO_2_) complexes, electron-withdrawing substituents had a beneficial effect for epoxidation activity. However, a generalization of this statement cannot be drawn, as at least complex [ReOCl(pyzOMe)_2_], equipped with an electron-donating −OMe substituent, also showed high epoxidation activity.^31^ Potentially, overall polarity has a bigger influence than electrophilicity of the metal center in these catalysts. More research is necessary to identify and understand all the structural properties that lead to more active catalysts.

**Fig. 7:**
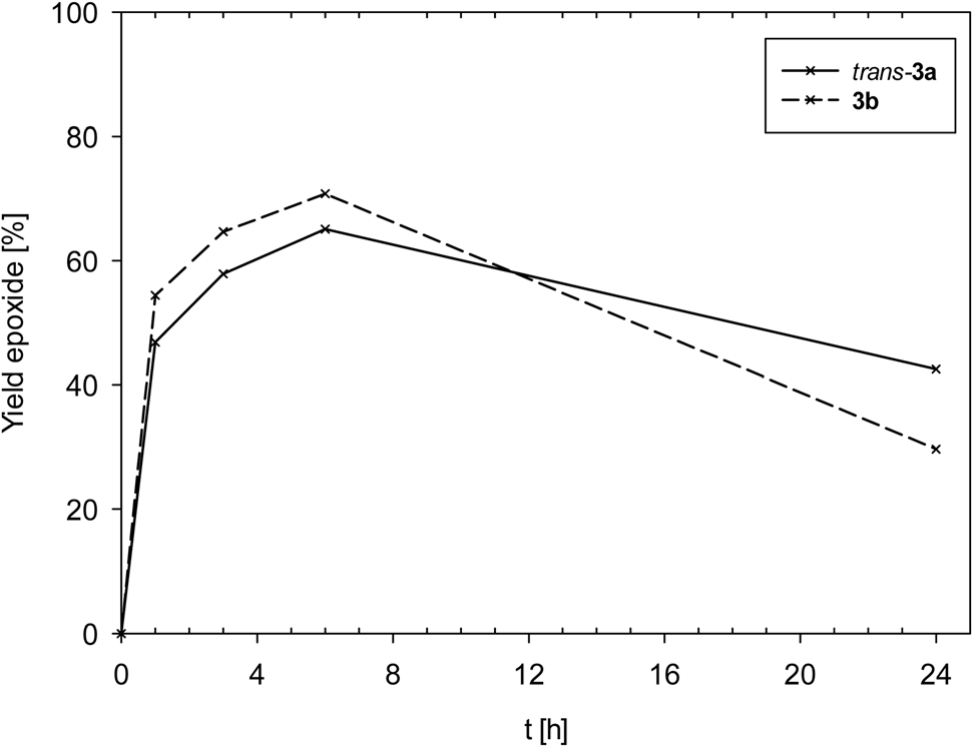
Comparison of epoxide yields of complexes *trans*-3a and 3b (lines between measured points are just optical guidelines without physical meaning).

Complexes 3a and 3b showed an overall very similar catalyst behavior of moderate activity. In contrast to 1, epoxide decomposition was observed upon longer reaction times. Both showed a high initial activity in the first hour (TOF_1h_ [h^−1^]: 3a = 47; 3b = 54), and a maximum conversion of substrate was reached after 6 h (TON: 3a = 65; 3b = 61). After 6 h though, for both complexes, the formed epoxide was consumed (Fig. 7), mainly by ring-opening to the diol, as observed by GC/MS. The overall similar catalyst profile of complexes 3a and 3b points to a lesser influence of the coordinated neutral ligand, SMe_2_ in 3a or OPPh_3_ in 3b. The observed ring-opening of the formed epoxide by 3a and 3b is not observed for complexes [ReOCl_2_(PPh_3_)(dmozR)] (R = H, OMe or NO_2_).^24^ As the PPh_3_ ligand should be a better Lewis base compared to SMe_2_ and OPPh_3_, the lesser overall chemical stability of 3a and 3b might lead to other metal containing decomposition products, thereby explaining the observed epoxide ring-opening by these decomposition products of 3a and 3b.

A review on the catalytic epoxidation activities of various [ReOX_2_(L_ON_)(PPh_3_)] complexes was published in 2020 by Wolff and Machura.^16^ Overall, the set of phenolate–pyrazole complexes [ReOCl_2_(pyzR)(PPh_3_)] still belong to the most active epoxidation catalysts, reaching TONs of >90.^31^ The catalytic profile of complexes 3a and 3b support the conclusion that the nature of the neutral ligand in such complexes (PPh_3_, OPPh_3_ or SMe_2_) is of lesser importance for catalytic activity. Overall, compared to the most active rhenium epoxidation catalyst, a variant of MTO, which reaches a TON of 20 000 in fluorinated solvents with added pyrazole,^35^ all the oxidorhenium(v) complexes are in general of far inferior activity.

### Oxyanion reduction

**Scheme 5:**
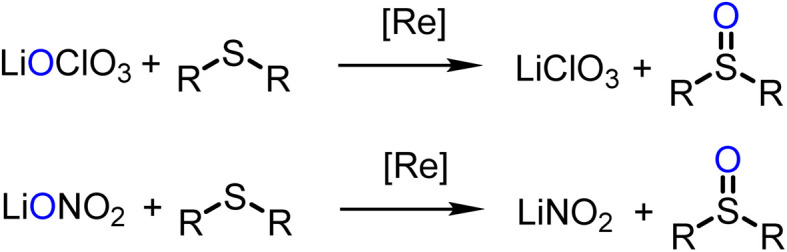
Comparison of first steps of catalytic perchlorate and nitrate reduction by oxidorhenium(v) complexes.

Experiments for catalytic reduction of perchlorate or nitrate were conducted under similar conditions (25 °C, CH_3_CN/H_2_O = 95/5 vol%). In perchlorate reduction, 3.2 mol% catalyst and 4 equiv. of SMe_2_ were used, in nitrate reduction experiments 10 mol% of catalyst and 5 equivalents of SMe_2_. Progress of the catalytic reaction was followed by the conversion of SMe_2_ to OSMe_2_*via*^1^H NMR spectroscopy. The onset of the catalytic reaction is the same for both substrates. A color change of initially green to light-orange is observed. The first, rate-determining reduction occurs for both substrates *via* a two-electron OAT mechanism (Scheme 5).

Complex 1 shows rather low activity in perchlorate reduction. At a 3.2 mol% catalyst loading, after 24 h only 12% of SMe_2_ was converted to OSMe_2_, as determined by ^1^H NMR spectroscopy (TON = 3.7). This low conversion is in line with the previous observation that electron-withdrawing substituents lower the catalytic perchlorate reduction activity. Parent complex [ReOCl(dmoz)_2_] showed a TON of 23, the nitro-substituted complex [ReOCl(dmozNO_2_)_2_] a TON of only 15 under the same experimental conditions.^23^ The original *N*,*N-trans* complex [ReOCl(oz)_2_] still shows the highest reported activity with a TON of 31 (full conversion).^20,23^

**Scheme 6:**
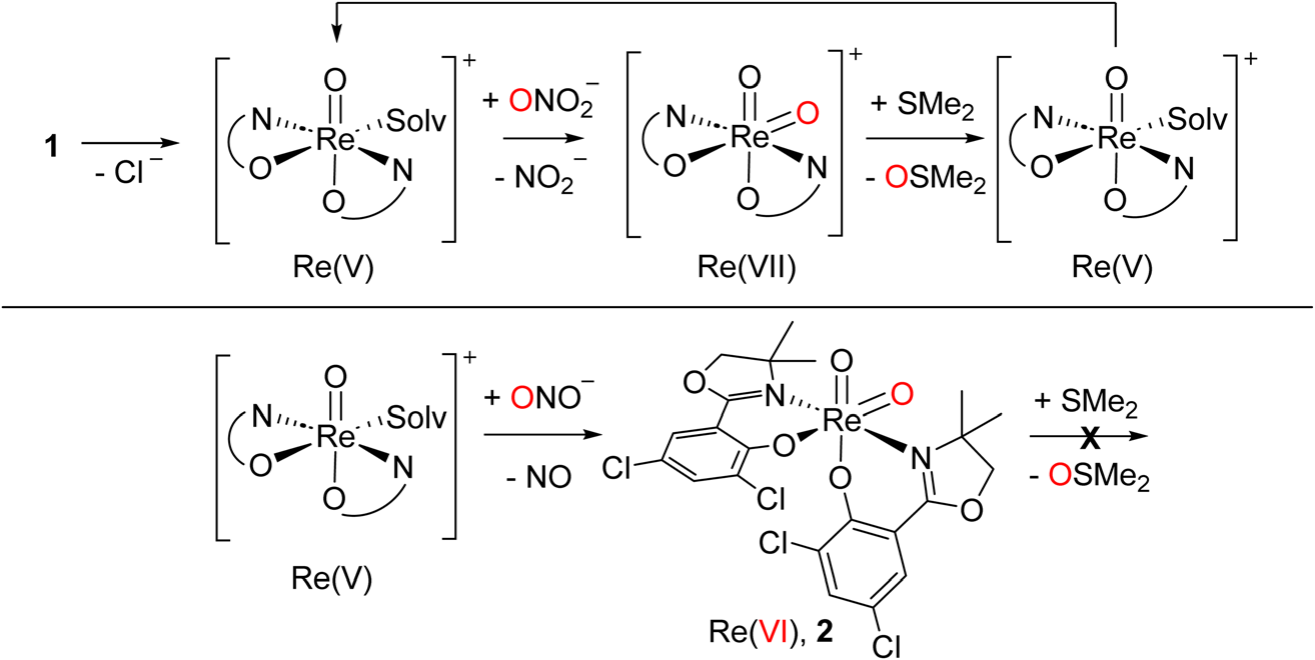
Top: catalytic nitrate reduction sequence starting with 1; bottom: stoichiometric single electron reduction of nitrite leading to 2 and NO; solv. = solvent.

Similar to perchlorate reduction, in nitrate reduction, the initial catalytic reduction of NO_3_^−^ to NO_2_^−^ (Scheme 6) occurs *via* a two-electron reduction of the substrate and an OAT to the Re center. The resulting dioxidorhenium(vii) cation [ReO_2_(L1)_2_]^+^ is then reduced by SMe_2_ regenerating the active oxidorhenium(v) species (Scheme 6, top). In contrast to chlorate ClO_3_^−^, the reduction of NO_2_^−^ occurs *via* a single-electron transfer yielding NO. Consequently, the resulting rhenium species is the singly-oxidized, paramagnetic and neutral dioxidorhenium(vi) complex [ReO_2_(L1)_2_]^0^ (2) (Scheme 6, bottom), consistent with the molecular solid state structure that was isolated from a nitrate reduction experiment. Complex 2 is not capable of performing OAT to the SMe_2_ acceptor. The same single electron oxidation to a neutral dioxidorhenium(vi) complex was observed for parent complex [ReOCl(dmoz)_2_].^26^ The reduction of nitrite and the chemistry of the reduction products are currently under investigation in our labs.

## Conclusions

Oxidorhenium(v) complexes equipped with an oxazoline–phenol ligand like HL1 remain a unique class of complexes capable of catalyzing OAT reactions with unusual, for other complexes highly unreactive substrates like perchlorate and nitrate. The additional benefit of high air and moisture stability makes this class of complexes an interesting target for further investigations towards oxyanion reduction. The electron-withdrawing dichloro-substituted ligand HL1 is another entry to the growing family of [ReOCl(dmozR)_2_] complexes. The electron-withdrawing nature of ligand HL1 proved again to have a beneficial effect on activity in catalytic epoxidation of cyclooctene, as complex 1 reached an unprecedented TON for oxidorhenium(v) complexes of 300. In contrast however, the same electron-withdrawing properties seem to result in a less active catalyst for perchlorate reduction. Here, un-substituted complex *N*,*N-trans* [ReOCl(oz)_2_] still shows the highest catalytic activities. In nitrate reduction, the isolation of paramagnetic dioxidorhenium(vi) complex 2 is in-line with the following sequence of reduction steps: catalytic 2e^−^ reduction of NO_3_^−^ to NO_2_^−^ followed by a 1e^−^ reduction of NO_2_^−^ to NO. The further fate of NO, a targeted synthesis as well as evaluation of the catalyst properties of complex 2 are subject to further investigations in our lab.

## Experimental

### General

The rhenium precursor [ReOCl_3_(OPPh_3_)(SMe_2_)] (P1) was prepared according to previously published methods.^36^ Full synthesis details of HL1 and can be found in the ESI.† Chemicals were purchased from commercial sources and used without further purification. NMR spectra were recorded with a Bruker Avance (300 MHz) instrument. Chemical shifts are given in ppm and are referenced to residual protons in the solvent. Coupling constants (*J*) are given in Hertz (Hz). Mass spectra were recorded with an Agilent 5973 MSD – Direct Probe using the EI ionization technique. Samples for infrared spectroscopy were measured on a Bruker Optics ALPHA FT-IR Spectrometer equipped with an ATR diamond probe head. GC-MS measurements were performed on an Agilent 7890 A with an Agilent 19091J–433 column coupled to a mass spectrometer type Agilent 5975C. Electrochemical measurements were performed under an inert N_2_ atmosphere in a glove box in dry acetonitrile (stored over molecular sieve) with a Gamry Instruments Reference 600 Potentiostat using a three-electrode setup. Platin was used as working electrode, Pt wire (99.99%) as supporting electrode; the reference electrode was a Ag wire immersed in a solution of 0.01 M AgNO_3_ and 0.1 M (NBu_4_)PF_6_ in CH_3_CN separated from the solution by a Vycor® tip. Supporting electrolyte used was (NBu_4_)PF_6_ (0.1 M). Elemental analyses were carried out using a Heraeus Vario Elementar automatic analyzer at the Graz University of Technology.

### Epoxidation of cyclooctene

A Heidolph Parallel Synthesizer 1 was used for all epoxidation experiments. In a typical experiment, 2–3 mg of catalyst (1 mol%) were dissolved in 0.5 mL CHCl_3_ and mixed with cyclooctene (1 equiv.) and 50 μL of mesitylene (internal standard) and heated to the respective reaction temperature (50 °C). Then the oxidant TBHP (3 equiv., 5.5 M in decane) was added. Aliquots for GC-MS (20 μL) were withdrawn at given time intervals, quenched with MnO_2_ and diluted with HPLC grade ethyl acetate. The reaction products were analysed by GC-MS (Agilent 7890 A with an Agilent 19091J-433 column coupled to a mass spectrometer type Agilent 5975C), and the epoxide produced from each reaction mixture was quantified *vs.* mesitylene as the internal standard. Catalytic experiments were only performed once. From previous experience, we estimate the experimental error of GC-MS measurements to be ± 5%.

### Syntheses of complexes 1, *trans*-3a and 3b

#### Synthesis of complex 1

Ligand HL1 (0.50 g, 1.92 mmol, 2 equiv.) and P1 (623 mg, 0.96 mmol, 1 equiv.) were mixed in 15 mL CH_3_CN. The greenish suspension was heated to refluxing conditions for a minimum of 16 h under which resulted in a clear green solution. After cooling the reaction solvent was removed completely to yield a deep-green oil. Repeated re-crystallization from CH_2_Cl_2_/heptane finally yielded 326 mg of crystalline 1 (0.43 mmol, 45% yield). ^1^H NMR (300 MHz, chloroform-d) *δ* 7.86 (d, *J* = 2.7 Hz, 1H, ar), 7.64 (d, *J* = 2.6 Hz, 1H, ar), 7.45 (d, *J* = 2.7 Hz, 1H, ar), 7.23 (d, *J* = 2.6 Hz, 1H, ar), 4.75 (d, *J* = 8.4 Hz, 1H, CH_2_), 4.69 (d, *J* = 8.3 Hz, 1H, CH_2_), 4.63 (d, *J* = 8.3 Hz, 1H, CH_2_), 4.42 (d, *J* = 8.4 Hz, 1H, CH_2_), 1.98 (s, 3H, CH_3_), 1.92 (s, 3H, CH_3_), 1.86 (s, 3H, CH_3_), 1.66 (s, 3H, CH_3_). ^13^C NMR (75 MHz, chloroform-d) *δ* 171.37, 168.58, 164.86, 159.46, 135.53 (ar. C–H), 135.40 (ar. C–H), 129.12 (ar. C–H), 128.48 (ar. C–H), 127.31, 124.59, 123.26, 121.85 (12 ar. C), 111.95, 111.22 (2 ipso-C, dmoz), 82.22 (CH_2_), 79.64 (CH_2_), 27.86 (CH_3_), 27.71 (CH_3_), 27.01 (CH_3_), 26.05 (CH_3_). ATR-IR (cm^−1^): 3083 (w), 2965 (w), 2899 (w), 1599 (C=N, m), 1437 (m), 1374 (m), 1310 (m), 1249 (m), 996 (s), 975 (Re=O, s), 872 (s), 780 (s), 731 (s), 446 (m); EI-MS (*m*/*z*): 756.4 (M^+^); UV (CH_2_Cl_2_) *λ*_max_, nm (*ε*): 655 (94); elemental analysis calculated for C_22_H_20_Cl_5_N_2_O_5_Re (755.9 g mol^−1^): C 34.96, H 2.67, N 3.71; found C 35.18, H 2.69, N 3.54.

#### Complex *trans*-3a

Ligand HL1 (1.00 g, 3.6 mmol, 2 equiv.) and P1 (1.16 g, 1.8 mmol, 1 equiv.) were mixed in CH_3_CN (20 mL) and stirred under refluxing conditions for 4 h, upon which a color change to green occurred. Upon cooling to rt, *trans*-3a precipitated as a dark-green, micro-crystalline solid. The solid was isolated and washed twice with small amounts of cold CH_3_CN. After drying, 157 mg of analytically pure *trans*-3a was obtained (264 μmol, 14%). ^1^H NMR (300 MHz, chloroform-d) *δ* 7.71 (d, *J* = 2.6 Hz, 1H, ar.), 7.36 (d, *J* = 2.6 Hz, 1H, ar.), 4.68 (s, 2H, CH_2_), 3.06 (s, 6H, S(CH_3_)_2_), 1.76 (s, 6H, 2*x* × CH_3_). ^13^C NMR (75 MHz, chloroform-d) *δ* 135.91, 129.61, 124.40, 123.23 (all ar.), 79.63 (CH_2_), 74.25 (CH_2_), 27.80 (S(CH_3_)_2_), 27.23 (some C obscured due to low solubility). ^1^H NMR (300 MHz, acetonitrile-d3) *δ* 7.75 (d, *J* = 2.6 Hz, 1H, ar.), 7.53 (d, *J* = 2.6 Hz, 1H, ar.), 4.75 (s, 2H, CH_2_), 3.02 (s, 6H, S(CH_3_)_2_), 1.66 (*s*, 6H, 2 × CH_3_). ATR-IR (cm^−1^): 2993, 2927, 1608 (C=N), 1576, 1546, 1446, 1396, 1332, 1254, 1216, 1141, 974 (Re=O), 869, 781, 737, 470; EI-MS (*m*/*z*): 594.9 (M^+^), 531.0 (M^+^–SMe_2_), 62.1 (SMe_2_); UV (CH_3_CN) *λ*_max_, nm (*ε*): 665 (28); elemental analysis calculated for C_13_H_16_N_4_Cl_4_NO_3_ReS (594.3 g mol^−1^): C 26.27, H 2.71, N 2.36, S 5.39; found C 26.22, H 2.62, N 2.33, S 5.25.

#### Complex 3b

After isolation of *trans*-3a the acetonitrile supernatant was slowly concentrated to approx. 4 mL of volume, leading to the precipitation of 3b as a green, micro-crystalline solid. Washing with small amounts of Et_2_O gave 310 mg of analytically pure product (382 μmol, 21%). ^1^H NMR (300 MHz, acetonitrile-d3) *δ* 7.70 (d, *J* = 2.6 Hz, 1H), 7.68–7.48 (m, 16H, OPPh_3_ and second aromatic proton), 4.72 (bs, 2H, CH_2_), 1.71 (s, 3H, CH_3_), 1.63 (s, 3H, CH_3_). ^13^C NMR (75 MHz, acetonitrile-d3) *δ* 137.01, 133.00, 132.97, 132.72, 132.59 (all P(C_6_H_5_)_3_), 129.73 (ar. C–H), 129.57 (P(C_6_H_5_)_3_), 80.77 (CH_2_), 26.88 (CH_3_), 26.41 (CH_3_) (some C obscured due to low solubility). ^31^P NMR (121 MHz, acetonitrile-d3) *δ* 27.11. ATR-IR (cm^−1^): 3092, 2974, 1599 and 1576 (C=N), 1436, 1378, 1310, 1250, 1129, 1115, 1080, 986 (Re=O), 962, 783, 721, 689, 534, 471; EI-MS (*m*/*z*): 774.1 (M^+^–Cl), 496.0 (M^+^–OPPh_3_,–Cl), 278.1 (OPPh_3_); UV (CH_3_CN) *λ*_max_, nm (*ε*): 695 (35); Elemental analysis calculated for C_29_H_25_N_4_Cl_4_NO_4_PRe (810.5 g mol^−1^): C 42.98, H 3.11, N 1.73; found C 43.12, H 3.22, N 1.77.

## Data availability

The data supporting this article has been included as part of the ESI.† Crystallographic data (excluding structure factors) for HL1, 1, 2, *trans-*3a, 3band 3cwere deposited with the Cambridge Crystallographic Data Center as supplementary publication no. 1913789 (HL1), 1913787 (1), 1995752 (2), 1854790 (*trans*-3a), 1854791 (3b) and 1854792 (3c). Copies of the data can be obtained free of charge on application to The Director, CCDC, 12 Union Road, Cambridge CB2 1EZ, UK [Fax: (internat.) +44–1223/336–033; E–mail: deposit@ccdc.cam.ac.uk].

## Conflicts of interest

There are no conflicts to declare.

## Supplementary Material

RA-014-D4RA07391F-s001

RA-014-D4RA07391F-s002
