# Supplementary material for: An oxidorhenium(v) complex with an electron-withdrawing ligand: benefits and drawbacks for a dual role catalyst
Source: RSC Adv. 2024 Dec 23;14(54):40058–68. doi: 10.1039/d4ra07391f (PMC11664244; doi:10.1039/d4ra07391f)
Supplement: RA-014-D4RA07391F-s001 [file RA-014-D4RA07391F-s001.pdf]

Supplementary Information (SI) for RSC Advances.
This journal is © The Royal Society of Chemistry 2024

Supporting information for

An oxidorhenium(V) complex with an electron-withdrawing ligand: benefits and drawbacks for a dual role catalyst

A. Gradenegger,[†] J. A. Schachner,*[,†] F. Belaj[†] and N. C. Mösch-Zanetti*[,†]
[†]Institute of Chemistry, University of Graz, Schubertstraße 1, 8010 Graz.

General. For experiments under microwave heating in the synthesis of ligand H**L1**, an Anton Paar Monowave 300 (850 W magnetron) with magnetic stirring was employed. Standard borosilicate glass reaction containers (G10, with snap caps) were used.

Synthesis of H**L1**.

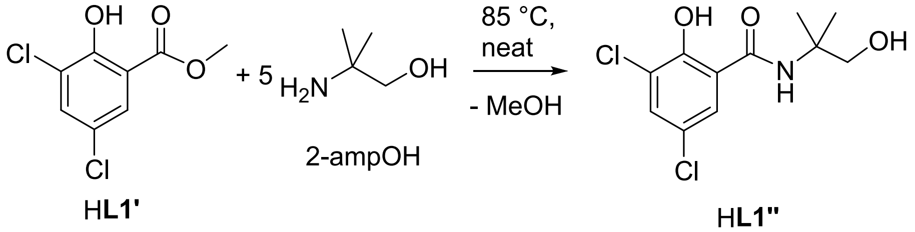

Scheme S1. Synthesis of ligand precursor H**L1''**. Synthesis of ligand precursor H**L1'** has been published.[1]

Synthesis of ligand precursor H**L1''**: 2.07 g (23.3 mmol, 5 equiv.) of 2-amino-2-methyl propanol (2-ampOH) was molten in a flask at 85 °C under stirring. Then H**L1'** (1.03 g, 4.66 mmol, 1 equiv.) was added slowly to the liquid amine. After 1h at 85 °C, the initial liquid reaction mixture had solidified and was cooled to room temperature. The mixture was dissolved in H_2O and acidified with 2M HCl, upon which the product H**L1''** precipitated. Extraction in Et_2O (3x 20 ml) yielded the pure product in quantitative yield as a white solid (1.29 g, 4.66 mmol, >95%). TLC (silica, EtOAC/cyclohexane 1/1) R_f = 0.41; ^{1}H NMR (300 MHz, chloroform-d) δ 12.59 (s, 1H, Ar-O*H*), 7.46 (s, 1H), 7.29 (s, 1H), 6.59 (bs, 1H, N*H*), 3.70 (s, 2H, -CH_2-), 1.44 (s, 6H, Me) (-CH_2-O*H* not visible due to deuterium scrambling); ATR-IR (cm^{-1}): 3480 (w), 3299 (w), 3076 (w), 3004 (w), 2979 (m), 2872 (m), 1643 (C=N, m), 1582 (m), 1551 (m), 1455 (m), 1344 (m), 1325 (m), 1253 (s), 1228 (s), 1198 (m), 1179(m), 1156(m), 1053 (m), 864(m), 739 (w), 653 (w), 478 (w), 419 (w); EI-MS (*m*/*z*): Only the M^+ peak for H**L1** is observed, due to ring closure under the experimental conditions for EI-MS (high vacuum, heating of sample).

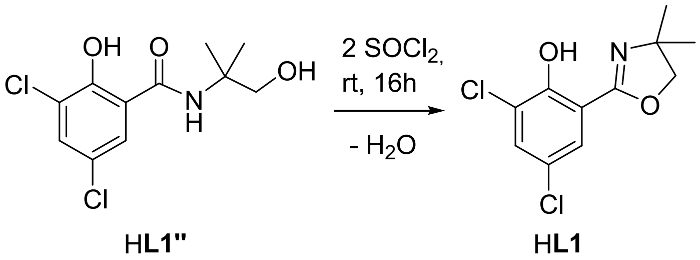

Scheme S2. Synthesis of ligand H**L1**.

Synthesis of ligand H**L1**: H**L1”** (4.72 g, 17.0 mmol, 1 equiv.) was suspended in 40 mL chloroform and thionyl chloride (2.5 mL, 34.1 mmol, 2 equiv.) was slowly added via syringe. After stirring overnight at rt the precipitated hydrochloride salt H**L1**·HCl was isolated by filtration and quenched with CH_2Cl_2/sat. $NaHCO_3$ to obtain H**L1** as an oily solid. Washing with cyclohexane gave an off-white solid (4.19 g, 16.1 mmol, >95%). TLC (silica, EtOAC/cyclohexane 1/1) $R_f = 0.58$; ^{1}H NMR (300 MHz, chloroform-d) δ 11.26 (s, 1H), 7.54 (d, J = 2.6 Hz, 1H), 7.45 (d, J = 2.6 Hz, 1H), 4.15 (s, 2H), 1.41 (s, 6H); ^{13}C NMR (75 MHz, Chloroform-d) δ 162.67, 154.75, 133.06, 126.06, 123.19, 122.56 (6 phenol-*C*), 112.68 (-*C*(O)=N-), 79.08 (-*C*H_2-), 67.53 (N-*C*(Me_2)-), 28.54 (-*C*H_3). ATR-IR (cm^{-1}): 3322 (w), 3091 (w), 2968 (m), 2928 (m), 2850 (m), 1633 (C=N, m), 1574 (m), 1454 (m), 1433 (m), 1356 (m), 1326 (m), 1279 (s), 1252 (s), 1175 (m), 967 (m), 809 (m), 764 (w), 718 (w), 557 (w); EI-MS (*m*/*z*): 259.1 (M^+).

Attempts of ligand synthesis via microwave heating. Each step of the above-described ligand syntheses was tested in addition with microwave heating with respect to a shortening of reaction times and steps. The thermal ring-closing without $SOCl_2$ has been published for other oxazoline ligands.[2] Hence, the direct synthesis of H**L1** from H**L1’** without the isolation of H**L1’’** was of specific interest. However, it was found that yields and purities of the respective products were always lower by microwave heating, compared to conventional heating.

Table S1. Reaction conditions of microwave heating and yields for H**L1’**.

entry	conditions[a]	yield H**L1’** [%]
1	100 °C, 10 min	< 10
2	150 °C, 10 min	37
3	150 °C, 20 min	51
4	150 °C, 40 min	57
5	200 °C, 20 min	decomposition
6	175 °C, 20 min	64
7	reflux, 12 h	92

[a] 0.5 g $HsalCl_2$, 32 µl H_2SO_4, 2.5 ml MeOH.

A summary of the results for the direct synthesis of H**L1** from methyl ester H**L1'** without the isolation of benzamide H**L1''** is shown in Table S2. The reaction mixture was tested by TLC for product distribution. Only where a single spot for ligand H**L1** was observed, the yield was determined.

Table S2. Reaction conditions of microwave heating and yields for H**L1**.

entry	equiv. 2-ampOH	solvent	conditions[a]	result
1	1	neat	200 °C, 20 min	several spots on TLC
2	1	EtOH	100 °C, 20 min	H**L1'** and H**L1''**
3	1	EtOH	150 °C, 20 min	H**L1'** and H**L1''**
4	1	EtOH	170 °C, 20 min	H**L1'**, H**L1''** and H**L1**
5	1	EtOH	150 °C, 240min	H**L1'**, H**L1''** and H**L1**
6	5	neat	150 °C, 10 min	H**L1''** and H**L1**
7	5	neat	150 °C, 20 min	H**L1''** and H**L1**
8	5	neat	200 °C, 20 min	de-halogenation
9	5	neat	180 °C, 90 min	H**L1**, yield: 20%
10	10	neat	170 °C, 20 min	H**L1''** and H**L1**
11	10	neat	180 °C, 20 min	H**L1''** and H**L1**
12	10	neat	180 °C, 40 min	H**L1**, yield: 54%
13	10	neat	100 °C, 15 min → 200 °C, 15 min	de-halogenation
14	10	neat	80 °C, 45 min → 180 °C, 45 min	yield after CC[b]: 64%

[a] 0.5 g H**L1'**, 2.5 ml EtOH (entries 2-5); [b] column chromatography.

As shown in Table S2, reaction temperatures of 200 °C led to decomposition of H**L1** (entries 1, 8 and 13). Judging from NMR data, a de-halogenation of the phenyl ring occurs at these temperatures, leading to the singly-chlorinated ligand. At temperatures of 180 °C, some product of H**L1** can be obtained (entries 9, 12 and 14). Considering that with our conventional synthesis high yields of H**L1** can be achieved without the need for column chromatography (CC), no further attempts for optimization by microwave heating were undertaken.

Synthesis of complexes 3a, 3b and isolation of 3c

When two equivalents of ligand H**L1** were heated to boiling with precursor **P1** in EtOH for four hours, only intractable mixtures of side-products where obtained under these conditions. This is in contrast to previously synthesized complexes [ReOCl(dmozR)$_2$] (R = H, OMe, NO_2).[3] When the reaction was attempted for four hours in boiling CH_3CN, a mixture of the two mono-ligated complexes [ReOCl$_2$(SMe$_2$)(**L1**)] (*trans*-**3a**) and [ReOCl$_2$(OPPh$_3$)(**L1**)] (**3b**) formed as the major products, but not the desired complex [ReOCl(**L1**)$_2$] (**1**) (Scheme S3). Obviously, the reaction time of four hours does not allow for the second **L1** equivalent to coordinate to the Re center. Upon cooling to room temperature, **3a** precipitated first from the reaction mixture as a green micro-crystalline solid in low yield of 14%. ^{1}H NMR spectra of **3a** only showed signals for one coordinated ligand moiety **L1**, together with the additional singlet peak for the SMe$_2$ ligand (Figure S8). In accordance to the recorded NMR spectra, MS-spectrometry found the M^+ peak at 594.9, further supporting the assignment of **3a** to be [ReOCl$_2$(SMe$_2$)(**L1**)]. The molecular structure of *trans*-**3a** was finally confirmed by single-crystal X-ray diffraction analysis (Figure S20), with the two chlorido ligands in a trans-orientation. The loss of the OPPh$_3$ instead of the SMe$_2$ ligand of **P1** is rarely observed.[4]

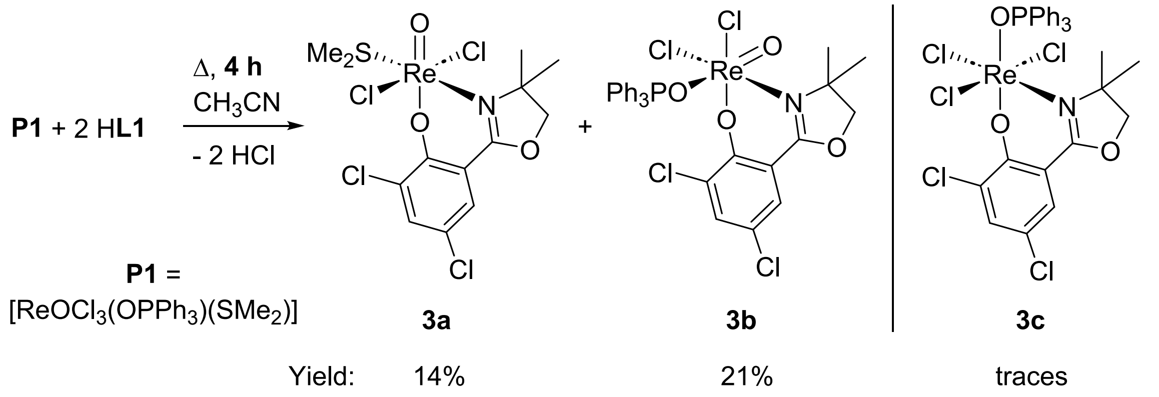

Scheme S3. Formation of oxidorhenium(V) complexes *trans*-**3a, 3b** and rhenium(IV) complex **3c** under short reaction times (4 h).

Upon further concentration of the acetonitrile supernatant, mono-ligated complex [ReOCl$_2$(OPPh$_3$)(**L1**)] (**3b**) precipitated, again as a green crystalline solid (yield 21%). Complex **3b** is mostly insoluble in CDCl$_3$, and just barely soluble enough in CD$_3$CN to obtain meaningful ^{1}H NMR spectra. A coordinated OPPh$_3$ ligand, appearing as the typical multiplet in the aromatic region, together with one coordinated **L1** moiety (Figure S10), indicated the composition of **3b** to be [ReOCl$_2$(OPPh$_3$)(**L1**)]. In the ^{31}P NMR spectrum, a peak at 27.11 ppm for **3b** could be assigned for the coordinated OPPh$_3$ ligand (Figure S12). The growth of single crystals suitable for X-ray diffraction analysis confirmed the molecular structure to be [ReOCl$_2$(OPPh$_3$)(**L1**)] (Figure S21). In literature, there are other examples for complexes of the

type [$ReOCl_2(OPPh_3)(LL)$] (LL = bidentate ligand),[5] and in a few cases by synthesis from **P1**.[4,6] The yields of *trans*-**3a** and **3b** are rather low (combined 35%). In an attempt to increase the yield of **3a/b**, a remaining supernatant acetonitrile solution was stored for several days in the freezer at -25 °C. Instead of expected green crystals of **3a/b**, bright red crystals formed in very small amounts. The red crystals were suitable for X-ray diffraction analysis, revealing this complex to be the rhenium(IV) complex [$ReCl_3(OPPh_3)$(**L1**)] (**3c**, Figure S22). As **3c** was never observed in crude reaction mixtures directly after synthesis of **3a/b**, we assume that **3c** is a decomposition product of **3a/b**. The reduction of oxidorhenium(V) complexes in the presence of oxidizable organic compounds, often P(III) ligands, has been observed before.[7] The formation of Re(IV)-$OPPh_3$ complexes in contrast is a rather rare occurence.[8] As the initial goal was to synthesize complex [ReOCl(**L1**)$_2$] (**1**), we did not further optimize the reaction conditions for a targeted synthesis of **3a** or **3b.**

Comparison of selected bond lengths and angles for 1 and 2 with previously published oxdiorhenium(V) and dioxidorhenium(VI) complexes

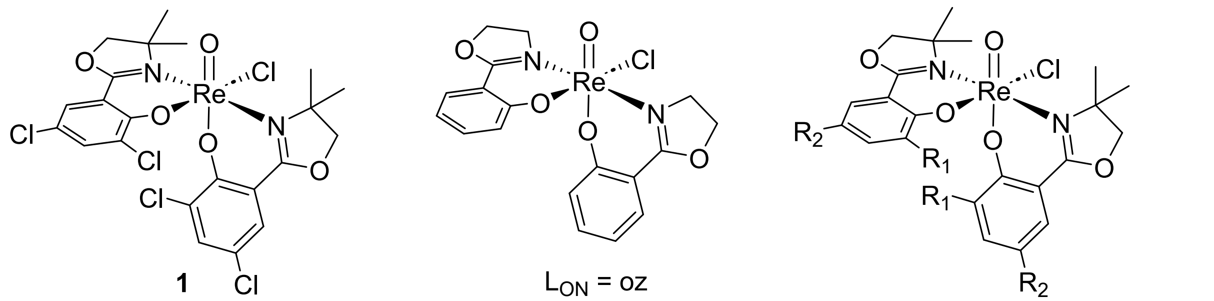

1 L_{ON} = oz

$R_1 = R_2$ = H, L_{ON} = dmoz
R_1 = OMe, R_2 = H, L_{ON} = dmoz(OMe)
R_1 = H, $R_2 = NO_2$, L_{ON} = dmoz(NO_2)

Figure S1. Oxidorhenium(V) complexes for structural comparison in Table S3

Table S3. Selected bond lengths [Å] of oxidorhenium(V) complex **1** with previously published oxidorhenium(V) complexes

ReOCl(L_{ON})$_2$	L_{ON} = **L1, 1**	L_{ON} = oz[9]	L_{ON} = dmoz[3]	L_{ON} = dmoz(OMe)[3]	L_{ON} = dmoz(NO_2)[3]
Re1=O1	1.700(5)	1.692(3)	1.682(6)	1.757(4)	1.719(4)
Re-Cl1	2.3960(17)	2.4093(10)	2.440(2)	2.400(2)	2.4087(18)
Re-O21	2.016(4)	2.001(3)	2.056(7)	1.987(4)	2.033(3)
Re1-O41	2.006(4)	2.007(3)	1.994(7)	1.999(4)	1.962(3)
Re-N13	2.106(5)	2.112(2)	2.203(5)	2.118(5)	2.205(3)
Re-N33	2.096(5)	2.064(3)	2.058(5)	2.096(4)	2.037(3)

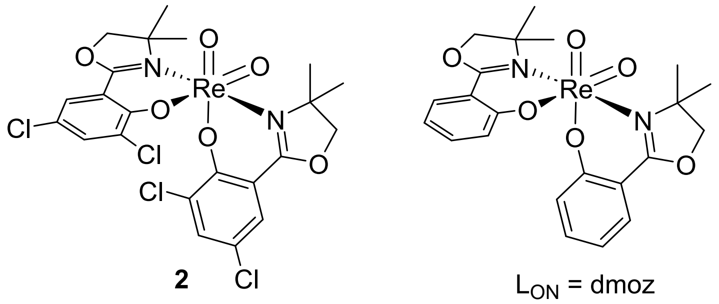

Figure S2. Dioxidorhenium(VI) complexes for structural comparison in Table S4

Table S4. Selected bond lengths [Å] of dioxidorhenium(VI) complex **2** with previously published dioxidorhenium(VI) complex

$ReO_2(L_{ON})_2$	L_{ON} = **L1**, **2**	L_{ON} = dmoz[10]
Re1=O1	1.7397(13)	1.745(6)
Re1=O2^i	1.7397(13)	1.732(6)
Re-O21	2.0680(12)	2.061(5)
Re1-O41^i	2.0680(12)	2.082(6)
Re-N13	2.0963(14)	2.098(7)
Re-N33^i	2.0963(14)	2.103(7)

^{i} equivalent atoms are generated by symmetry (two-fold rotation axis)

NMR spectra

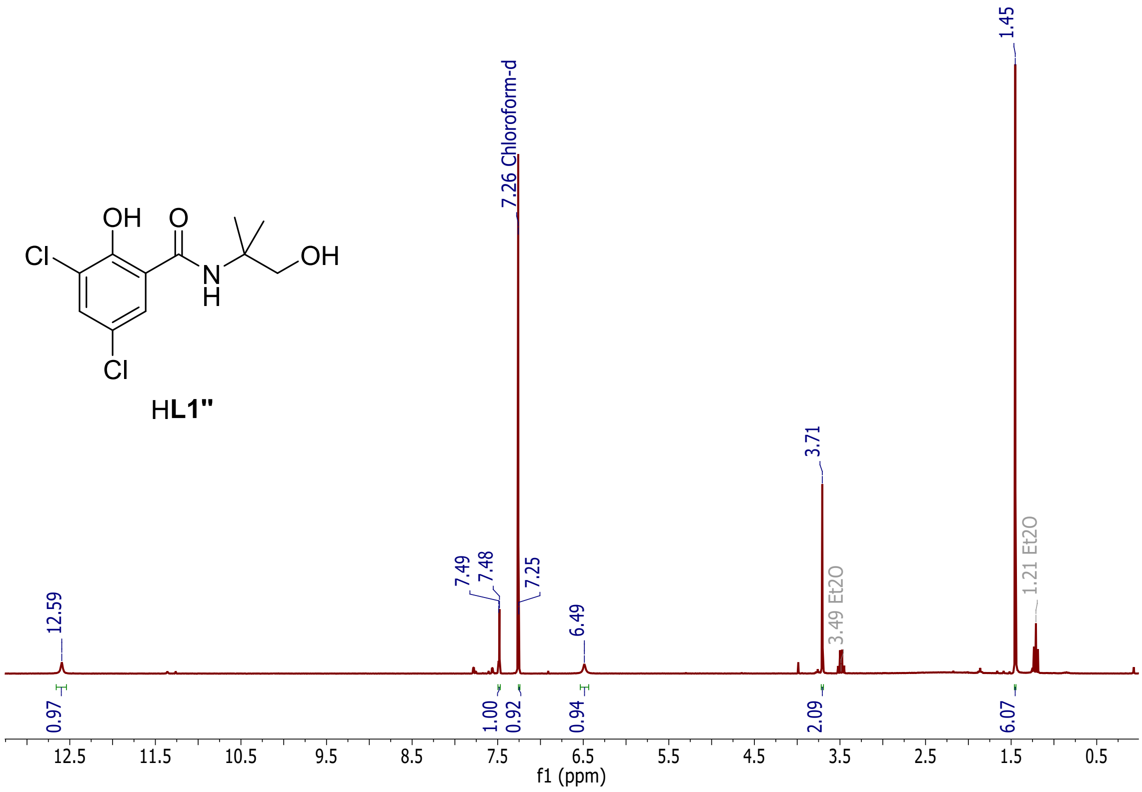

Figure S3. ^{1}H NMR spectrum of H**L1''** ($CDCl_3$).

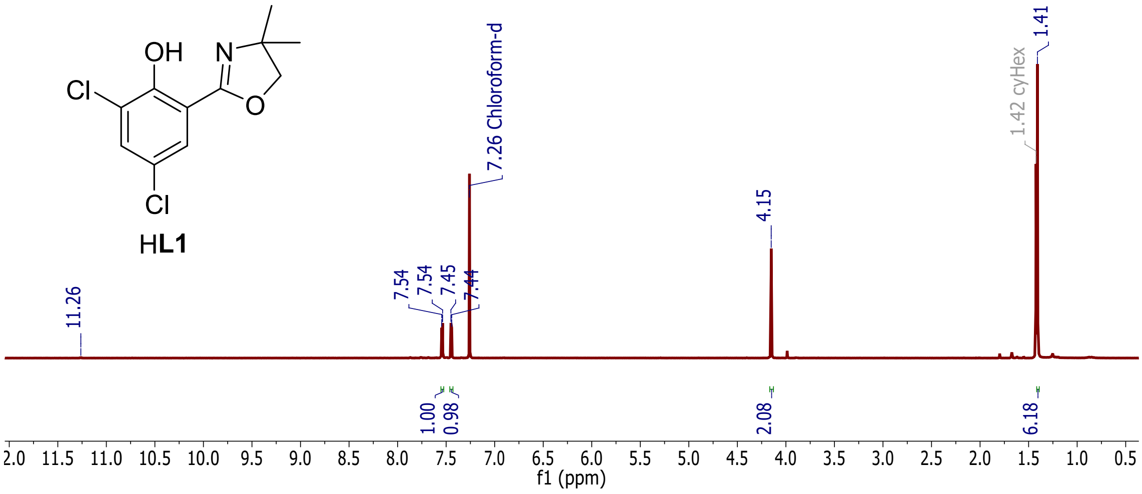

Figure S4. ^{1}H NMR spectrum of H**L1** ($CDCl_3$).

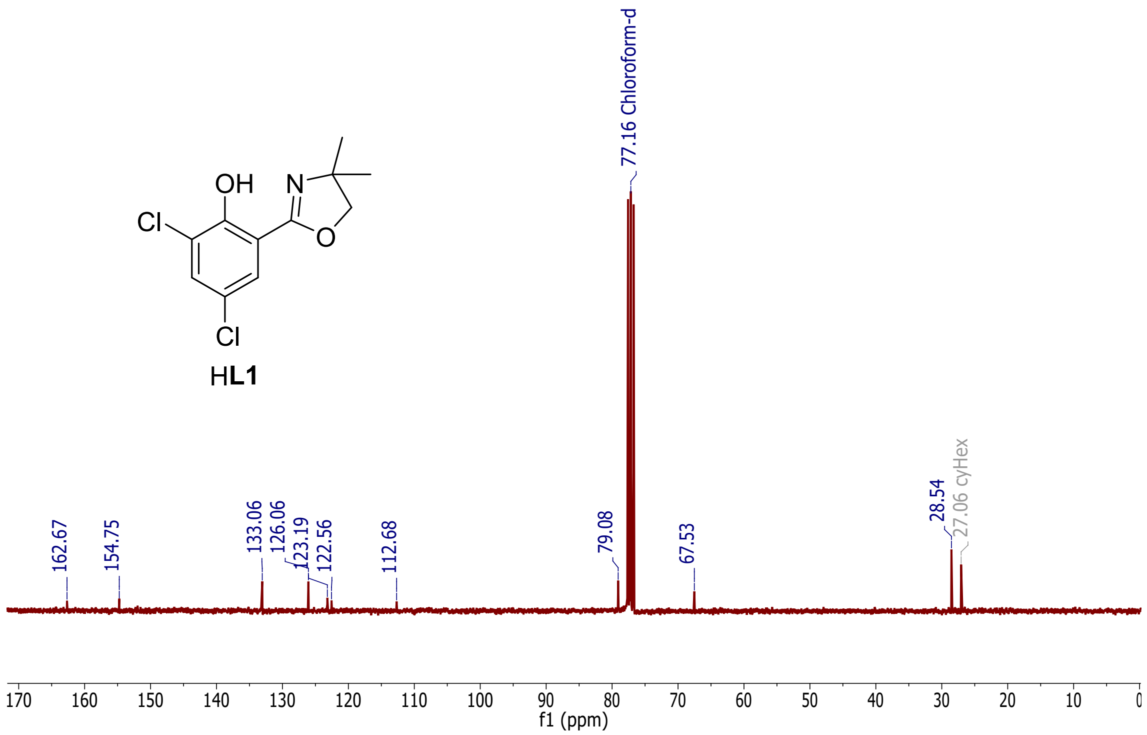

Figure S5. ^{13}C NMR spectrum of H**L1** ($CDCl_3$).

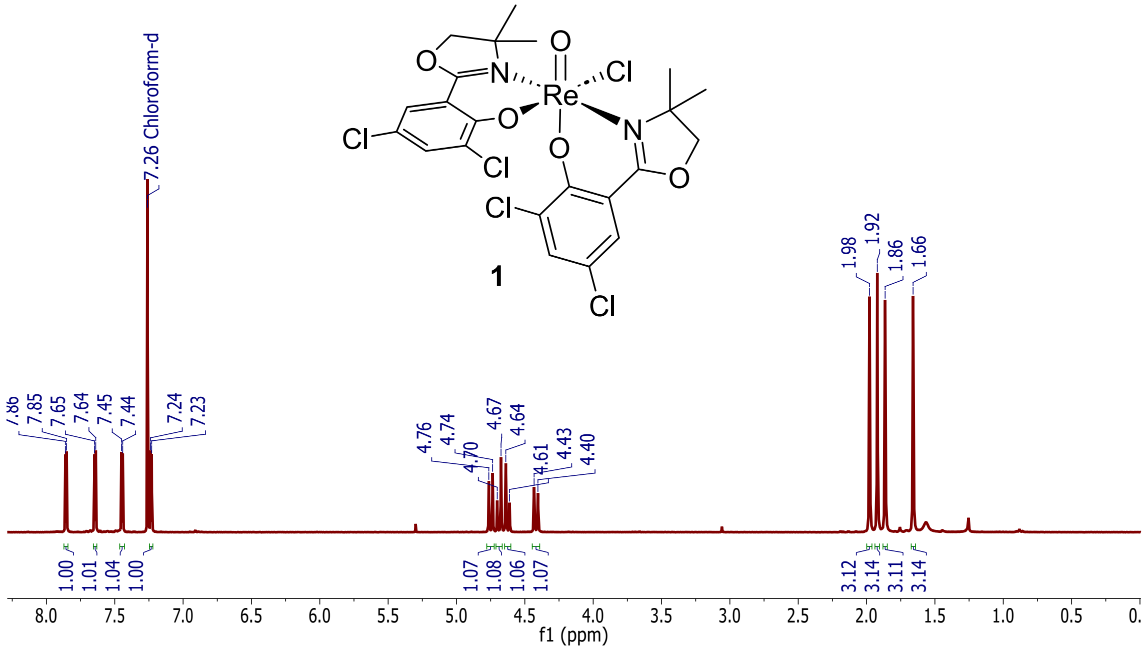

Figure S6. ^{1}H NMR spectrum of **1** ($CDCl_3$).

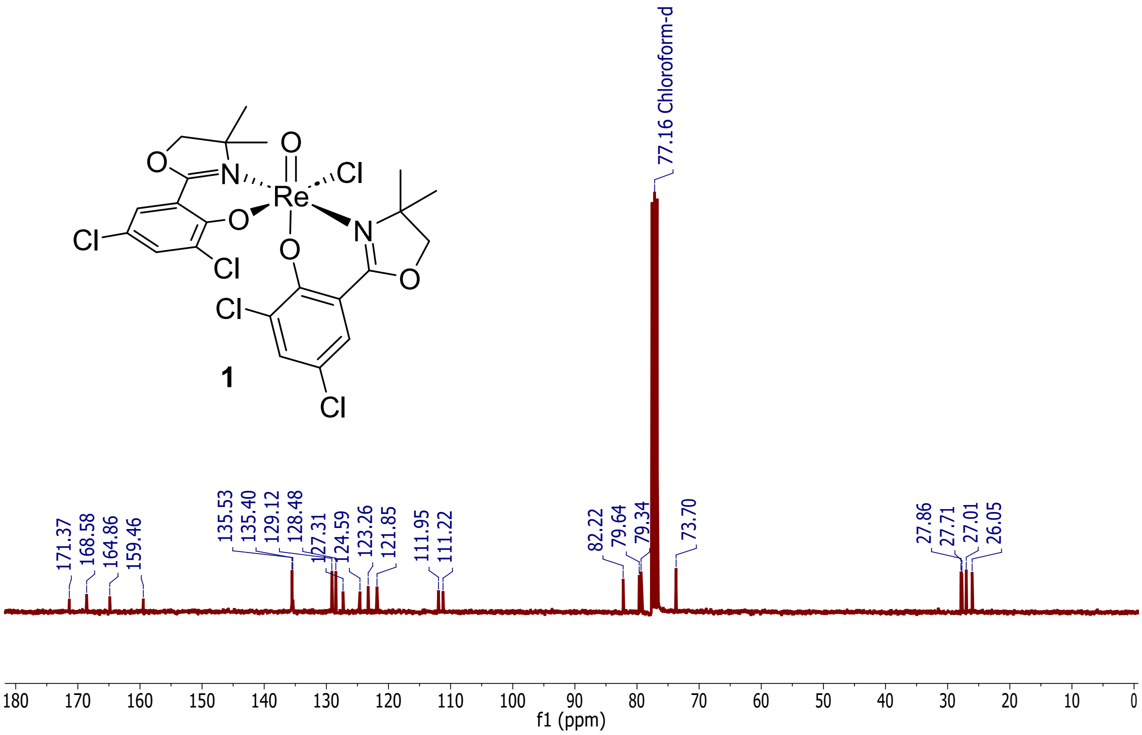

Figure S7. ^{13}C NMR spectrum of **1** ($CDCl_3$).

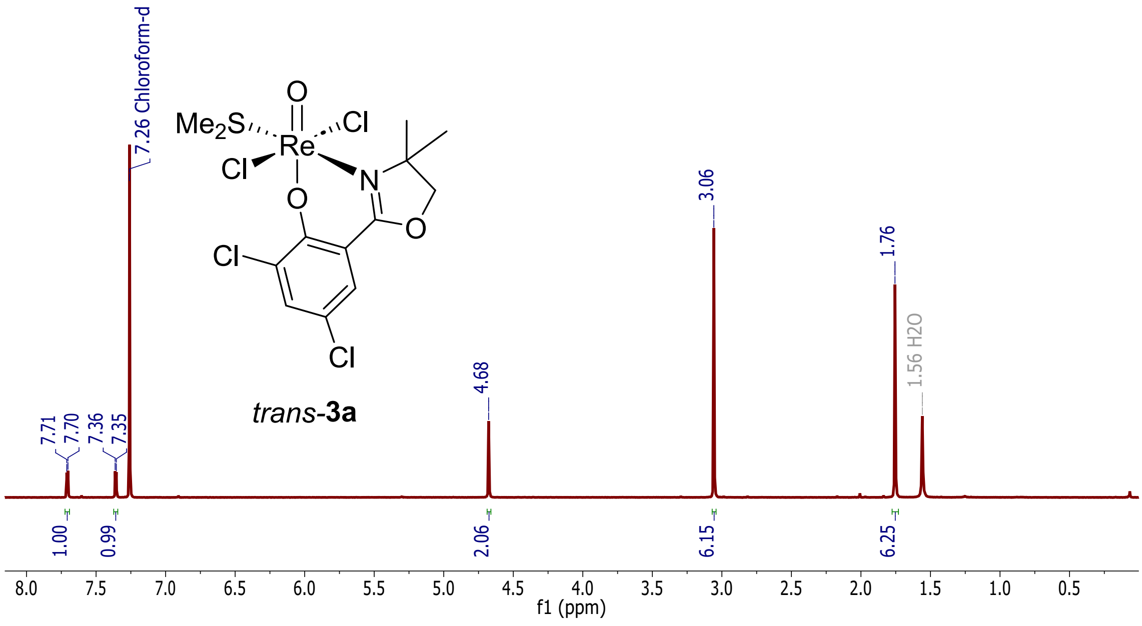

Figure S8. ^{1}H NMR spectrum of *trans*-**3a** ($CDCl_3$).

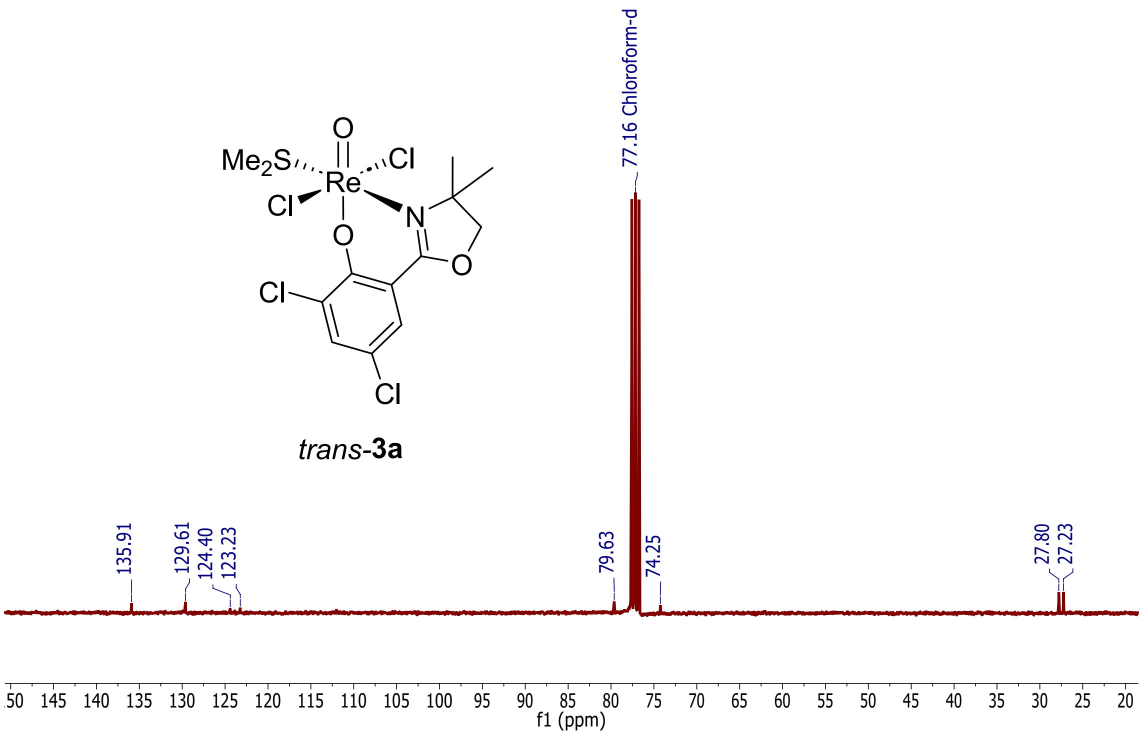

Figure S9. ^{13}C NMR spectrum of *trans*-**3a** ($CDCl_3$).

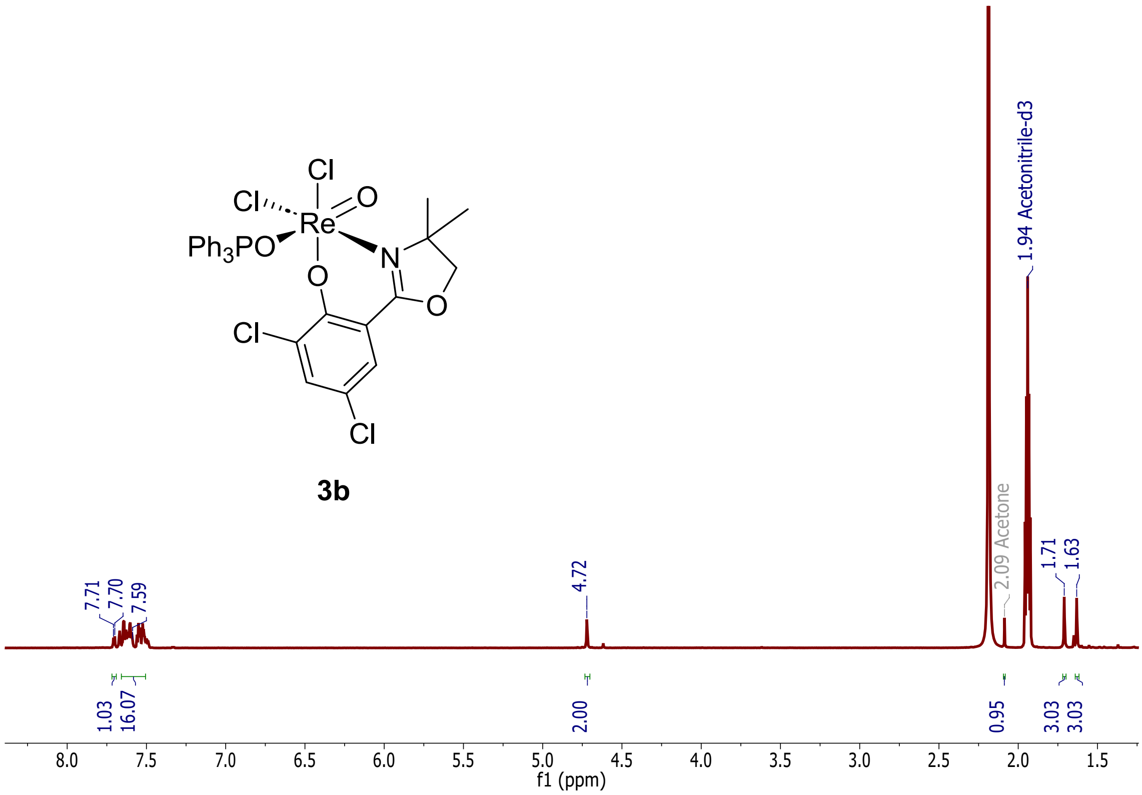

Figure S10. ^{1}H NMR spectrum of **3b** (CD_3CN).

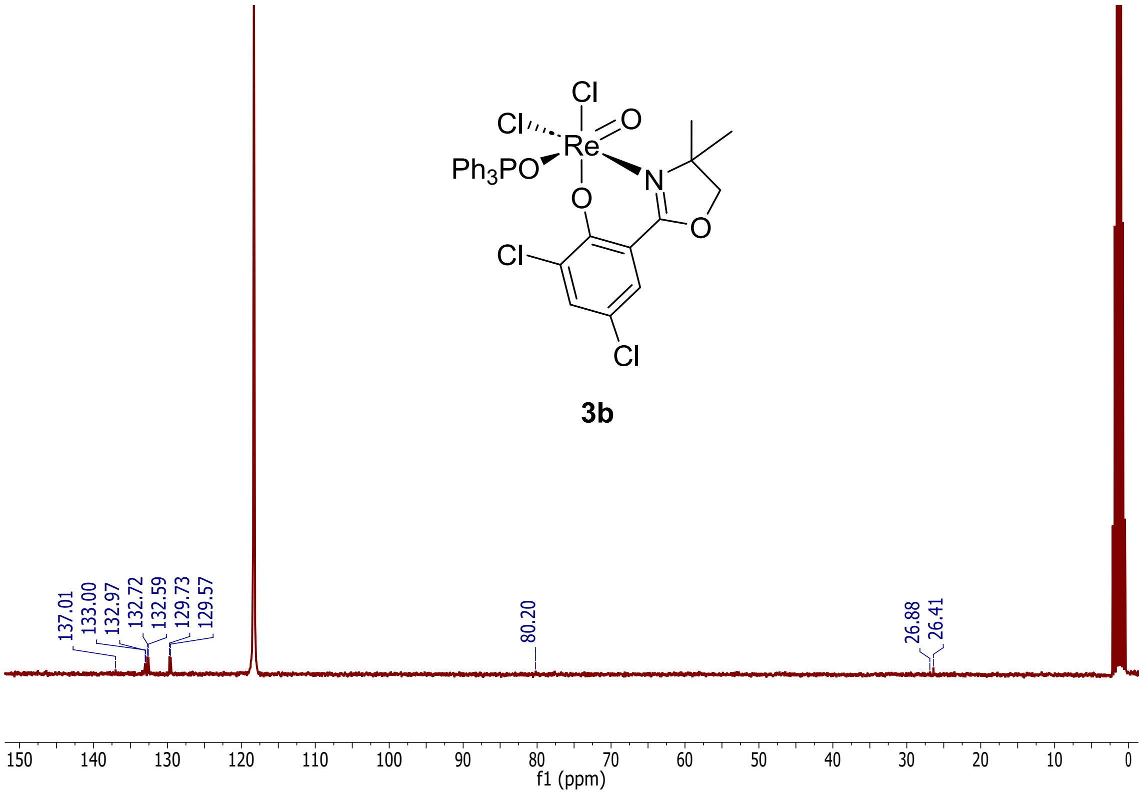

Figure S11. ^{13}C NMR spectrum of **3b** (CD_3CN).

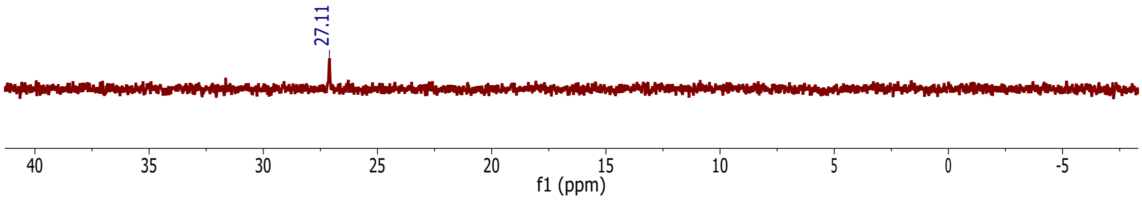

Figure S12. ^{31}P NMR of **3b** (CD_3CN).

Cyclic voltammetry. Electrochemical measurements were performed under an inert N_2 atmosphere in a glove box in dry acetonitrile (stored over molecular sieve) with a Gamry Instruments Reference 600 Potentiostat using a three electrode setup. Platin was used as working electrode, Pt wire (99.99%) as supporting electrode; the reference electrode was a Ag wire immersed in a solution of 0.01 M $AgNO_3$ and 0.1 M $(NBu_4)PF_6$ in CH_3CN separated from the solution by a Vycor® tip. Analyte solutions were near 1 mM with $(NBu_4)PF_6$ used as supporting electrolyte (0.1 M). The currents I_p were normalized by the actual concentrations to allow better comparability. All measurements were referenced to the ferrocenium (Fc^+)/ferrocene (Fc) couple.

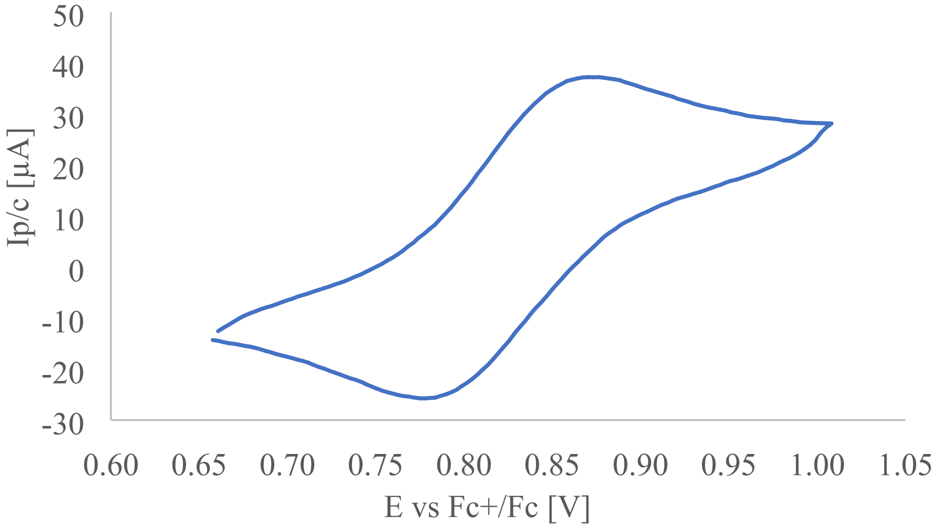

Figure S13. Cyclic voltammogram of **1**; $E_{1/2}$ = 0.85 V.

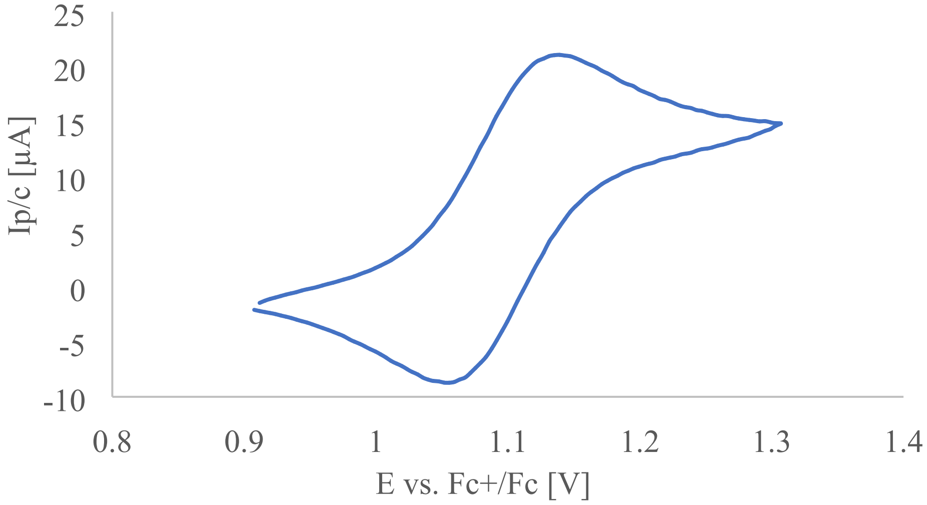

Figure S14. Cyclic voltammogram of *trans*-**3a**; $E_{1/2}$ = 1.10 V.

Crystal structure determinations. All the single crystal measurements were performed on a Bruker APEX-II CCD diffractometer at 100 K using Mo K_{α} radiation with a wavelength of 0.71073Å from an Incoatec microfocus sealed tube equipped with a multilayer monochromator. Absorption corrections were made semi-empirically from equivalents. The structures were solved by direct methods (SHELXS-97)[11] and refined by full-matrix least-squares techniques against F^2 (SHELXL-2014/6)[12]. A weighting scheme of $w = 1/[\sigma^2(F_o^2)+(aP)^2+bP]$ where $P = (F_o^2+2F_c^2)/3$ was used. The absolute configurations of H**L1** and of *trans*-**3a** were established by anomalous dispersion effects in the diffraction measurements on the crystals. The non-hydrogen atoms were refined with anisotropic displacement parameters without any constraints. The position of the H atom of the OH group of H**L1** was taken from a difference Fourier map, the O–H distance was fixed to 0.84Å, and this H atom was refined with an individual isotropic displacement parameter without any constraints to the bond angles. The H atoms of the phenyl rings were put at the external bisectors of the C–C–C angles at C–H distances of 0.95Å and common isotropic displacement parameters were refined for the H atoms of the same ring. The H atoms of the CH_2 groups were refined with common isotropic displacement parameters for the H atoms of the same group and idealized geometries with approximately tetrahedral angles and C–H distances of 0.99Å. The H atoms of the methyl groups were refined with common isotropic displacement parameters for the H atoms of the same group and idealized geometries with tetrahedral angles, enabling rotations around the C–C bonds, and C–H distances of 0.98Å. Crystal data, data collection parameters and structure refinement details are given in the following. Further refinement information, structure and bonding parameters, SHELXL .res and .hkl files are given in the deposited CIF file which is available free of charge from The Cambridge Crystallographic Data Centre (CCDC 1913789, 1913787, 1854790-1854792, 1995752).

Table S5. Crystallographic data and structure refinement details of H**L1** and **1**.

**Crystal data**	H**L1**	**1**
CIF data code	JSJZ15	JSJZ2B
Empirical formula	$C_{11}H_{11}Cl_2NO_2$	$C_{22}H_{20}Cl_5N_2O_5Re$
Formula weight	260.11	755.85
Crystal description	plate, colourless	plate, green
Crystal size	0.36 x 0.14 x 0.03mm	0.31 x 0.27 x 0.04mm
Temperature	100K	100K
Crystal system	monoclinic	monoclinic
Space group	C c	P 2_1/n
a	7.4471(9)Å	11.0483(15)Å
b	23.852(4)Å	12.4028(17)Å
c	6.8468(8)Å	37.115(5)Å
α		
β	107.136(7)°	93.108(6)°
γ		
Volume	1162.2(3)Å^3	5078.4(12)Å^3
Z	4	8
Calc. density	1.487Mg/m^3	1.977Mg/m^3
F(000)	536	2928
Linear absorption coefficient μ	0.542mm^{-1}	5.349mm^{-1}
Max. and min. transmission	1.000 and 0.613	1.000 and 0.640
Unit cell determination	2.99° < Θ < 28.73°	2.47° < Θ < 26.84°
Reflections used	4813	9774
**Data collection**		
Θ range for data collection	2.99 to 29.99°	1.73 to 26.00°
Reflections collected / unique	17577 / 3384	25840 / 9991
Significant unique reflections	2846 with I > 2σ(I)	7907 with I > 2σ(I)
R(int), R(sigma)	0.0857, 0.0675	0.0378, 0.0676
Completeness to $Θ_{max}$	99.9%	99.9%
**Refinement**		
Data / parameters / restraints	3384 / 155 / 3	9991 / 655 / 0
Goodness-of-fit on F^2	1.008	1.007
Final R indices [I > 2σ(I)]	R1 = 0.0365,<br>wR2 = 0.0713	R1 = 0.0447,<br>wR2 = 0.1110
R indices (all data)	R1 = 0.0505,<br>wR2 = 0.0762	R1 = 0.0546,<br>wR2 = 0.1147
Weighting scheme param. a, b	0.0257, 0.0000	0.0650, 0.0000
Largest Δ/σ in last cycle	0.001	0.002
Largest diff. peak and hole	0.277 and -0.258e/Å^3	1.794 and -1.740e/Å^3
**CCDC no.**	1913789	1913787

Table S7. Crystallographic data and structure refinement details of **2** and *trans*-**3a**.

**Crystal data**	**2**	*trans*-**3a**
CIF data code	JSJZ21	JS76IV
Empirical formula	$C_{22}H_{20}Cl_4N_2O_6Re$	$C_{13}H_{16}Cl_4NO_3ReS$
Formula weight	736.40	594.33
Crystal description	block, orange	block, green
Crystal size	0.28 x 0.13 x 0.09mm	0.24 x 0.24 x 0.15mm
Temperature	100K	100K
Crystal system	orthorhombic	monoclinic
Space group	P c c n	C c
a	16.202(3)Å	8.3972(12)Å
b	16.335(3)Å	32.451(5)Å
c	18.805(3)Å	6.6411(11)Å
α		
β		94.071(5)°
γ		
Volume	4976.9(15)Å^3	1805.1(5)Å^3
Z	8	4
Calc. density	1.966Mg/m^3	2.187Mg/m^3
F(000)	2856	1136
Linear absorption coefficient μ	5.355mm^{-1}	7.450mm^{-1}
Max. and min. transmission	1.000 and 0.593	0.747 and 0.455
Unit cell determination	2.50° < Θ < 40.71°	2.51° < Θ < 35.74°
Reflections used	9744	9365
**Data collection**		
Θ range for data collection	1.77 to 40.00°	2.51 to 35.00°
Reflections collected / unique	77915 / 15402	16795 / 6728
Significant unique reflections	10723 with I > 2σ(I)	6295 with I > 2σ(I)
R(int), R(sigma)	0.0542, 0.0424	0.0417, 0.0475
Completeness to Θ_{max}	99.9%	99.9%
**Refinement**		
Data / parameters / restraints	15402 / 330 / 0	6728 / 218 / 2
Goodness-of-fit on F^2	1.018	1.034
Final R indices [I > 2σ(I)]	R1 = 0.0283, wR2 = 0.0605	R1 = 0.0298, wR2 = 0.0663
R indices (all data)	R1 = 0.0501, wR2 = 0.0686	R1 = 0.0349, wR2 = 0.0690
Weighting scheme param. a, b	0.0217, 2.4883	0.0381, 0.0000
Largest Δ/σ in last cycle	0.002	0.003
Largest diff. peak and hole	1.776 and -1.583e/Å^3	1.847 and -1.429e/Å^3
**CCDC no.**	1995752	1854790

Table S8. Crystallographic data and structure refinement details of **3b** and **3c**.

**Crystal data**	**3b**	**3c**
CIF data code	JS76	JS76V
Empirical formula	$C_{29}H_{25}Cl_4NO_4PRe$	$C_{29}H_{25}Cl_5NO_3PRe$
Formula weight	810.47	829.92
Crystal description	plate, green	plate, orange
Crystal size	0.25 x 0.17 x 0.07mm	0.16 x 0.08 x 0.04mm
Temperature	100K	100K
Crystal system	monoclinic	triclinic
Space group	P 2_1/n	P -1
a	11.6314(5)Å	9.9619(18)Å
b	20.2311(9)Å	10.0674(18)Å
c	12.6581(6)Å	17.161(3)Å
α		73.649(5)°
β	101.269(2)°	76.698(5)°
γ		71.136(6)°
Volume	2921.2(2)$Å^3$	1544.3(5)$Å^3$
Z	4	2
Calc. density	1.843Mg/m^3	1.785Mg/m^3
F(000)	1584	810
Linear absorption coefficient μ	4.617mm^{-1}	4.451mm^{-1}
Max. and min. transmission	1.000 and 0.719	1.000 and 0.624
Unit cell determination	2.40° < Θ < 30.49°	2.35° < Θ < 23.47°
Reflections used	8513	5384
**Data collection**		
Θ range for data collection	1.92 to 30.00°	2.19 to 26.00°
Reflections collected / unique	26705 / 8522	27366 / 6063
Significant unique reflections	6802 with I > 2σ(I)	4977 with I > 2σ(I)
R(int), R(sigma)	0.0505, 0.0558	0.0893, 0.0721
Completeness to $Θ_{max}$	99.9%	99.9%
**Refinement**		
Data / parameters / restraints	8522 / 370 / 0	6063 / 370 / 0
Goodness-of-fit on F^2	1.014	1.029
Final R indices [I > 2σ(I)]	R1 = 0.0343, wR2 = 0.0732	R1 = 0.0359, wR2 = 0.0630
R indices (all data)	R1 = 0.0512, wR2 = 0.0789	R1 = 0.0534, wR2 = 0.0685
Weighting scheme param. a, b	0.0305, 2.1365	0.0174, 1.0201
Largest Δ/σ in last cycle	0.004	0.001
Largest diff. peak and hole	1.982 and -1.204$e/Å^3$	0.973 and -1.207$e/Å^3$
**CCDC no.**	1854791	1854792

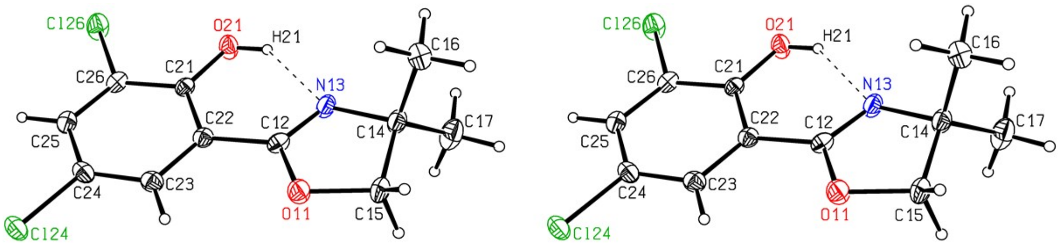

Figure S15**.** Stereoscopic ORTEP[13] plot of H**L1** showing the atomic numbering scheme. The probability ellipsoids are drawn at the 50% probability level. The H atoms are drawn with arbitrary radii. The hydrogen bond is indicated by a dashed line.

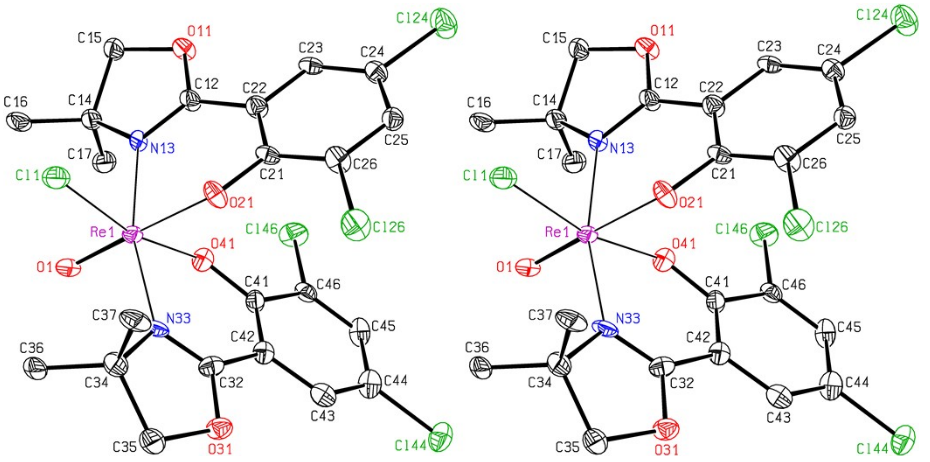

Figure S16. Stereoscopic ORTEP[13] plot of complex **A** of **1** showing the atomic numbering scheme. The probability ellipsoids are drawn at the 50% probability level. The H atoms were omitted for clarity.

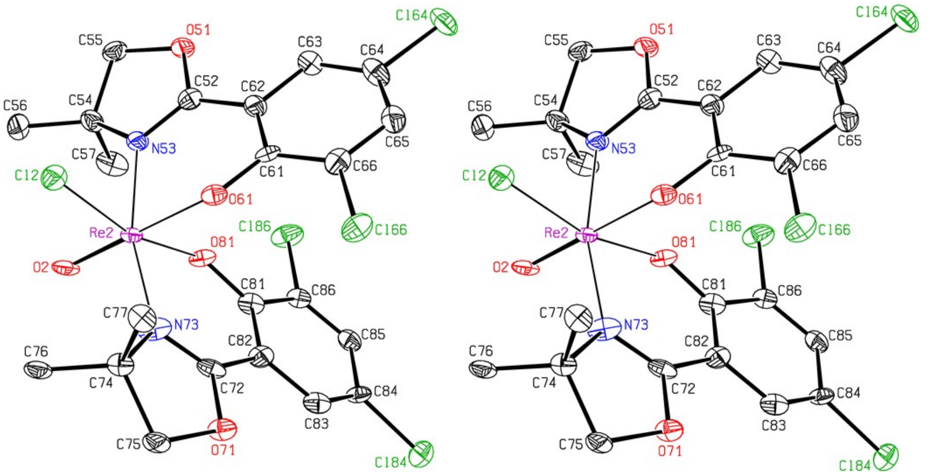

Figure S17. Stereoscopic ORTEP[13] plot of complex **B** of **1** showing the atomic numbering scheme. The probability ellipsoids are drawn at the 50% probability level. The H atoms were omitted for clarity.

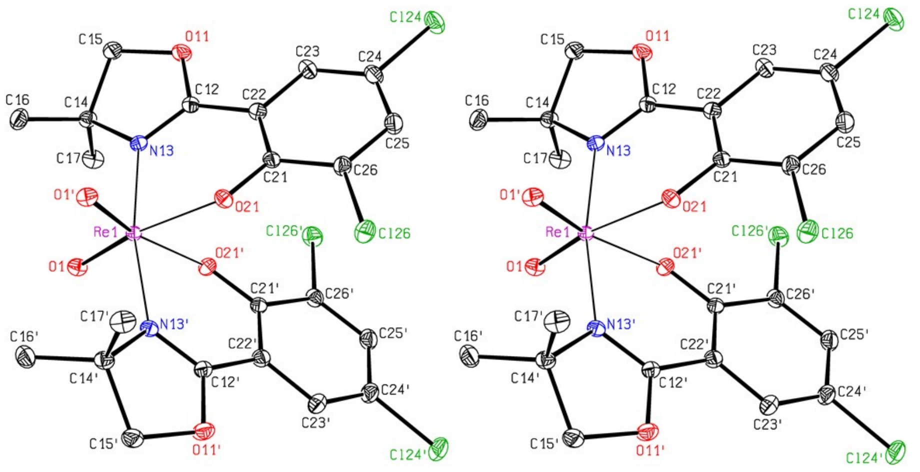

Figure S18. Stereoscopic ORTEP[13] plot of complex **A** of **2** showing the atomic numbering scheme. The probability ellipsoids are drawn at the 50% probability level. The H atoms were omitted for clarity.

Table S8. Selected bond lengths [Å] and angles [°] for **1**.

Re1-O1	1.700(5)	O1-Re1-O21	177.7(2)	O2-Re2-O61	179.66(19)
Re1-O21	2.016(4)	N13-Re1-N33	164.7(2)	N53-Re2-N73	164.7(2)
Re1-O41	2.006(4)	O41-Re1-Cl1	167.46(13)	O81-Re2-Cl2	166.14(13)
Re1-N13	2.106(5)	C12-N13-C14	106.2(5)	C52-N53-C54	105.6(5)
Re1-N33	2.096(5)	C12-N13-Re1	126.6(4)	C52-N53-Re2	126.7(4)
Re1-Cl1	2.3960(17)	C14-N13-Re1	125.6(4)	C54-N53-Re2	125.8(4)
Re2-O2	1.690(5)	C21-O21-Re1	131.8(4)	C61-O61-Re2	130.8(4)
Re2-O61	2.012(4)	C32-N33-C34	107.9(5)	C72-N73-C74	105.9(5)
Re2-O81	1.985(4)	C32-N33-Re1	123.7(4)	C72-N73-Re2	123.3(4)
Re2-N53	2.107(5)	C34-N33-Re1	128.1(4)	C74-N73-Re2	130.1(4)
Re2-N73	2.070(5)	C41-O41-Re1	127.9(4)	C81-O81-Re2	129.4(4)
Re2-Cl2	2.4103(17)				

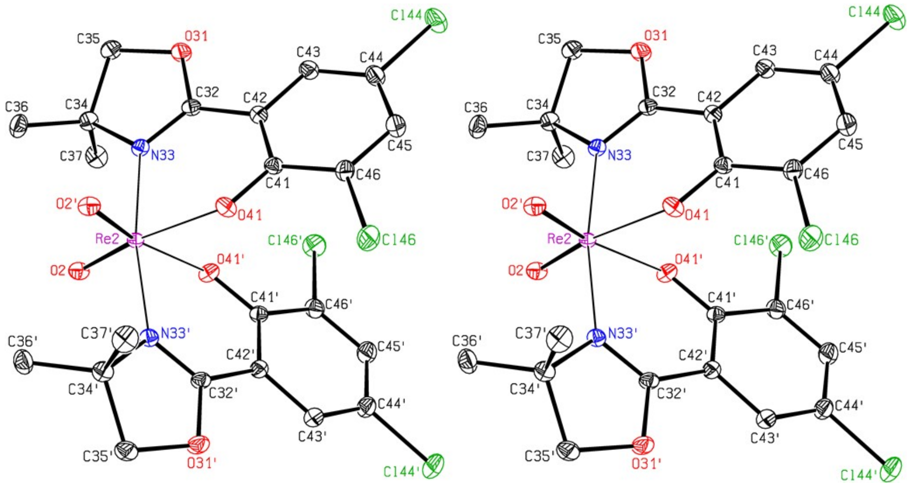

Figure S19. Stereoscopic ORTEP[13] plot of complex **B** of **2** showing the atomic numbering scheme. The probability ellipsoids are drawn at the 50% probability level. The H atoms were omitted for clarity.

Table S9. Selected bond lengths [Å] and angles [°] for **2**.

Re1-O1	1.7397(13)	O1-Re1-O21	164.36(5)	O2-Re2-O41	164.32(6)
Re1-O21	2.0680(12)	N13-Re1-N13$^{i)}$	169.04(7)	N33-Re2-N33$^{ii)}$	170.21(7)
Re1-N13	2.0963(14)	C12-N13-C14	108.18(13)	C32-N33-C34	107.60(14)
Re2-O2	1.7342(13)	C12-N13-Re1	125.37(11)	C32-N33-Re2	125.81(11)
Re2-O41	2.0689(12)	C14-N13-Re1	126.08(10)	C34-N33-Re2	126.44(11)
Re2-N33	2.0984(15)	C21-O21-Re1	128.44(11)	C41-O41-Re2	128.54(11)

Symmetry transformations used to generate equivalent atoms:

$^{i)}$ 1/2-x, 1/2-y, z $^{ii)}$ 3/2-x, 1/2-y, z

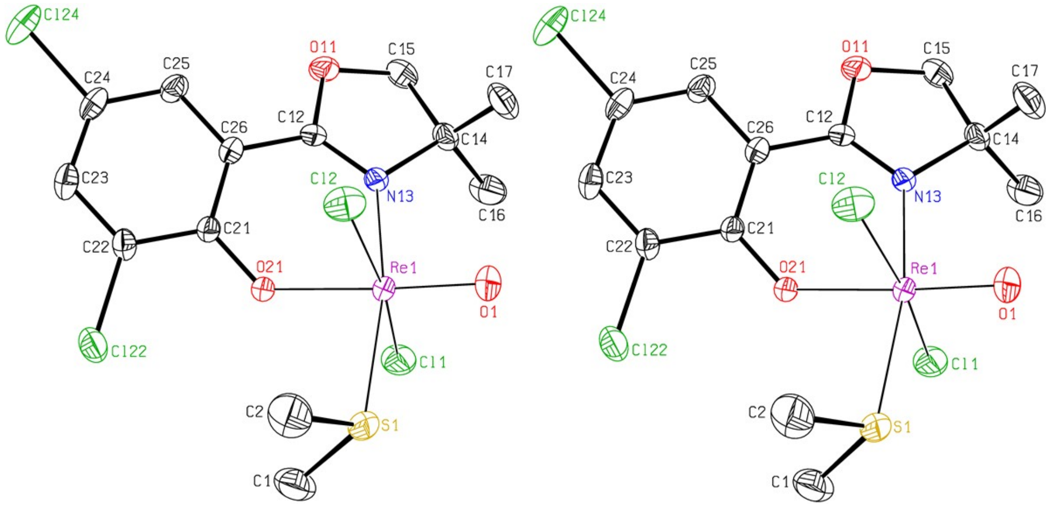

Figure S20. Stereoscopic ORTEP[13] plot of *trans*-**3a** showing the atomic numbering scheme. The probability ellipsoids are drawn at the 50% probability level. The H atoms were omitted for clarity.

Table S10. Selected bond lengths [Å] and angles [°] for *trans*-**3a**.

Re1-O1	1.677(4)	O1-Re1-O21	176.68(19)
Re1-N13	2.113(5)	Cl1-Re1-Cl2	171.43(5)
Re1-O21	1.989(3)	N13-Re1-S1	169.08(11)
Re1-Cl1	2.3846(15)	C12-N13-C14	107.7(4)
Re1-Cl2	2.4057(14)	C12-N13-Re1	125.8(3)
Re1-S1	2.4238(15)	C14-N13-Re1	125.8(3)
S1-C2	1.799(8)	C21-O21-Re1	131.0(3)
S1-C1	1.803(8)	C1-S1-C2	99.5(4)
		C1-S1-Re1	107.0(3)
		C2-S1-Re1	110.1(3)

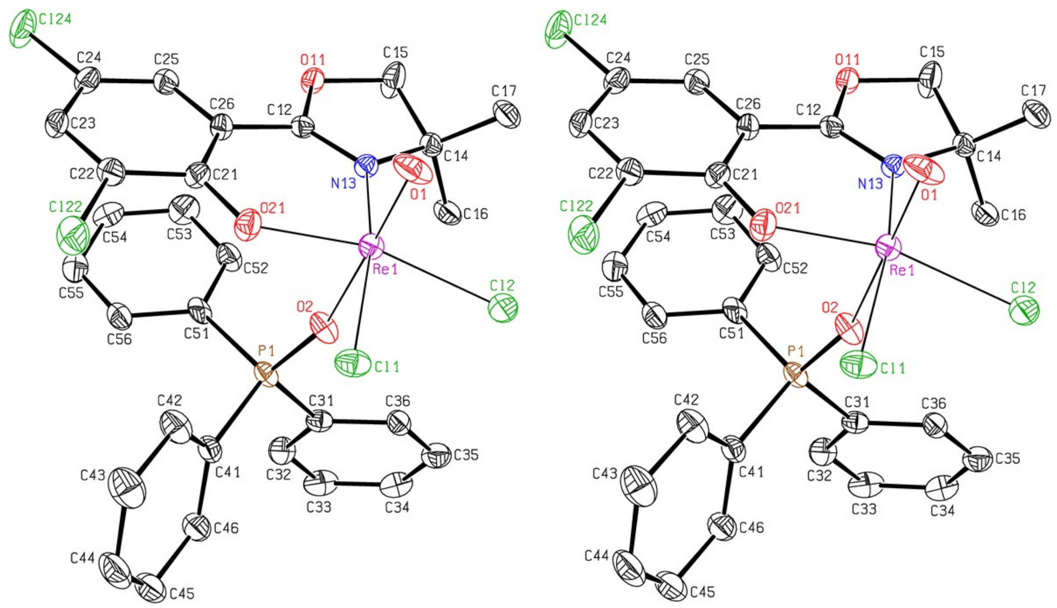

Figure S21. Stereoscopic ORTEP[13] plot of **3b** showing the atomic numbering scheme. The probability ellipsoids are drawn at the 50% probability level. The H atoms were omitted for clarity.

Table S11. Selected bond lengths [Å] and angles [°] for **3b**.

Re1-O1	1.692(3)	O1-Re1-O2	173.90(12)
Re1-O2	2.141(3)	N13-Re1-Cl1	167.70(9)
Re1-N13	2.107(3)	O21-Re1-Cl2	164.76(9)
Re1-O21	1.998(2)	Cl1-Re1-Cl2	87.75(3)
Re1-Cl1	2.3670(9)	C12-N13-C14	108.5(3)
Re1-Cl2	2.3858(10)	C12-N13-Re1	122.9(2)
O2-P1	1.510(3)	C14-N13-Re1	128.5(2)
		C21-O21-Re1	129.9(2)
		P1-O2-Re1	159.66(17)

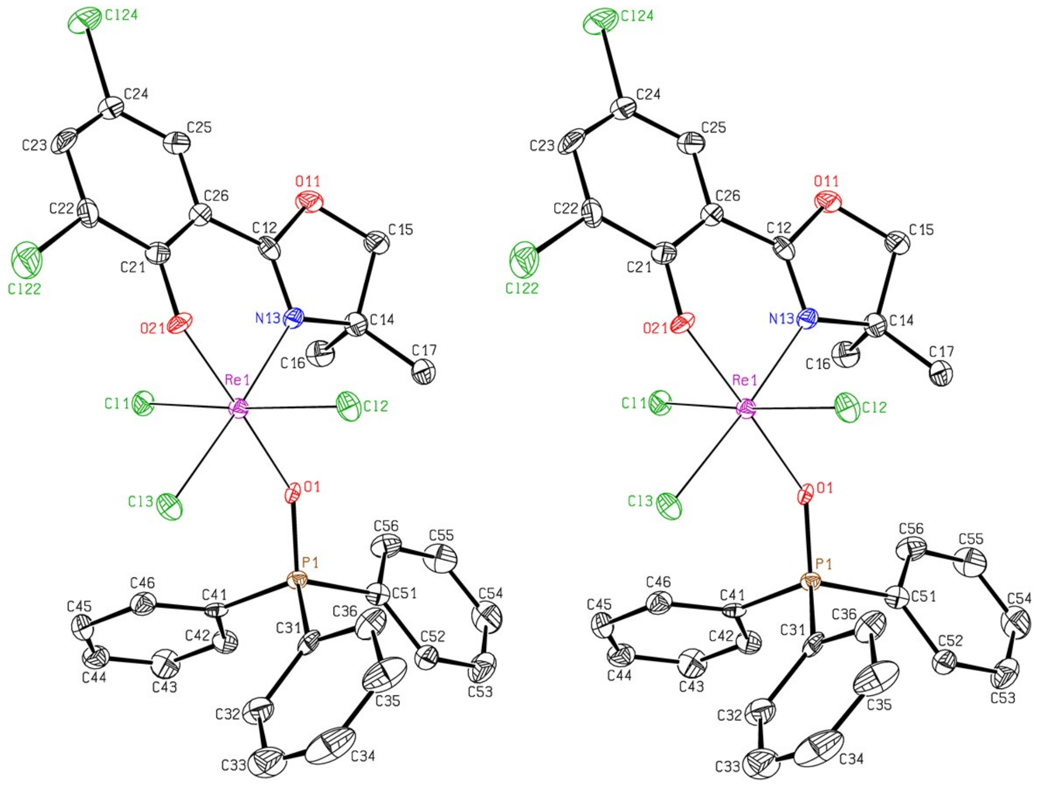

Figure S22. Stereoscopic ORTEP[13] plot of **3c** showing the atomic numbering scheme. The probability ellipsoids are drawn at the 50% probability level. The H atoms were omitted for clarity.

Table S12. Selected bond lengths [Å] and angles [°] for **3c**.

Re1-O1	2.053(3)	O1-Re1-O21	177.55(13)
Re1-N13	2.105(4)	N13-Re1-Cl3	175.08(11)
Re1-O21	1.974(3)	Cl1-Re1-Cl2	175.67(4)
Re1-Cl1	2.3443(12)	C12-N13-C14	107.0(4)
Re1-Cl2	2.3522(12)	C12-N13-Re1	123.5(3)
Re1-Cl3	2.3268(12)	C14-N13-Re1	129.1(3)
O1-P1	1.534(3)	C21-O21-Re1	127.6(3)
		P1-O1-Re1	144.71(19)

1. A. Itai, S. Muto, R. Tokuyama, H. Fukasawa and T. Yanase, *Preparation of thiazinone and benzothiazinone derivatives and analogs as inhibitors of 11β-hydroxysteroid dehydrogenase type I for treatment of diabetes: European Patent Application* WO2009038064A1(EP2208728A1), 2010.
2. L. N. Pridgen and G. Miller, *J. Heterocycl. Chem.*, 1983, **20**, 1223–1230.
3. J. A. Schachner, B. Berner, F. Belaj and N. C. Mösch-Zanetti, *Dalton Trans.*, 2019, **48**, 8106-8115.
4. L. Hansen, E. Alessio, M. Iwamoto, P. A. Marzilli and L. G. Marzilli, *Inorg. Chim. Acta*, 1995, **240**, 413–417.
5. a) U. Abram, A. Voigt, R. Kirmse, K. Ortner, R. Hübener, R. Carballo and E. M. Vázquez-López, *Z. Anorg. Allg. Chem.*, 1998, **624**, 1662–1668; b) T. Ohashi, Y. Miyashita, Y. Yamada, K. Fujisawa and K. Okamoto, *Bull. Chem. Soc. Jpn.*, 2003, **76**, 1199–1205; c) S. Fortin and A. L. Beauchamp, *Inorg. Chem.*, 2000, **39**, 4886–4893; d) B. Machura, A. Świtlicka, M. Wolff and J. Kusz, *Struct. Chem.*, 2009, **20**, 911–918;
6. a) K. A. Nolin, R. W. Ahn, Y. Kobayashi, J. J. Kennedy-Smith and F. D. Toste, *Chem. Eur. J.*, 2010, **16**, 9555–9562; b) K. A. Nolin, R. W. Ahn and F. D. Toste, *J. Am. Chem. Soc.*, 2005, **127**, 12462–12463; c) B. G. Das, R. Nallagonda, D. Dey and P. Ghorai, *Chem. Eur. J.*, 2015, **21**, 12601–12605; d) A. Skarżyńska and M. Siczek, *Polyhedron*, 2008, **27**, 1930–1936; e) B. Machura, M. Wolff, I. Gryca and R. Kruszynski, *Polyhedron*, 2012, **40**, 93–104;
7. a) B. Machura, A. Jankowska, R. Kruszynski, J. Kłak and J. Mroziński, *Polyhedron*, 2006, **25**, 2663–2672; b) S. R. Lane, N. Sisay, B. Carney, S. Dannoon, S. Williams, H. P. Engelbrecht, C. L. Barnes and S. S. Jurisson, *Dalton Trans.*, 2011, **40**, 269–276; c) D. A. Rotsch, K. M. Reinig, E. M. Weis, A. B. Taylor, C. L. Barnes and S. S. Jurisson, *Dalton Trans.*, 2013, **42**, 11614; d) J. Liu, J. K. Choe, Y. Wang, J. R. Shapley, C. J. Werth and T. J. Strathmann, *ACS Catal.*, 2015, **5**, 511–522; e) B. K. Dirghangi, M. Menon, A. Pramanik and A. Chakravorty, *Inorg. Chem.*, 1997, **36**, 1095–1101; f) S. Das and A. Chakravorty, *Eur. J. Inorg. Chem.*, 2006, 2285–2291;
8. a) J. Y. Kim, Y. J. Ji, H.-J. Ha and H. K. Chae, *Bull. Korean Chem. Soc.*, 2003, **24**, 504–506; b) U. Abram, R. Carballo, S. Cabaleiro, S. Garcia-Fontán and E. M. Vázquez-López, *Polyhedron*, 1999, **18**, 1495–1499;
9. J. A. Schachner, B. Terfassa, L. M. Peschel, N. Zwettler, F. Belaj, P. Cias, G. Gescheidt and N. C. Mösch-Zanetti, *Inorg. Chem.*, 2014, **53**, 12918–12928.

10. J. A. Schachner, F. Wiedemaier, N. Zwettler, L. M. Peschel, A. D. Boese, F. Belaj and N. C. Mösch-Zanetti, *J. Catal.*, 2021, **397**, 108-115.
11. G. M. Sheldrick, *Acta Crystallogr., Sect. A: Found. Crystallogr.*, 2008, 112–122.
12. G. M. Sheldrick, *Acta Crystallogr., Sect. C: Cryst. Struct. Chem.*, 2015, 3–8.
13. C. K. Johnson, *ORTEP: A FORTRAN Thermal-Ellipsoid Plot Program for Crystal Structure Illustrations: ONRL Report #3794*, Oak Ridge National Laboratory, 1965.
